# Phosphorylation of endothelial histone H3.3 serine 31 by PKN1 links flow-induced signaling to proatherogenic gene expression

**DOI:** 10.1038/s44161-024-00593-y

**Published:** 2025-01-08

**Authors:** Young-June Jin, Guozheng Liang, Rui Li, ShengPeng Wang, Mohamad Wessam Alnouri, Mette Bentsen, Carsten Kuenne, Stefan Günther, Yang Yan, Yongxin Li, Nina Wettschureck, Stefan Offermanns

**Affiliations:** 1 Department of Pharmacology, Max Planck Institute for Heart and Lung Research, Bad Nauheim, Germany; 2 Department of Cardiovascular Medicine, The First Affiliated Hospital of Xi’an Jiaotong University, Xi’an, China; 3 Bioinformatics and Deep Sequencing Platform, Max Planck Institute for Heart and Lung Research, Bad Nauheim, Germany; 4 Department of Cardiovascular Surgery, The First Affiliated Hospital of Xi’an Jiaotong University, Xi’an, China; 5 Centre for Molecular Medicine, Medical Faculty, JW Goethe University Frankfurt, Frankfurt, Germany; 6 Cardiopulmonary Institute (CPI), Frankfurt, Germany; 7 German Center for Cardiovascular Research (DZHK), Rhine-Main site, Frankfurt and Bad Nauheim, Germany

**Keywords:** Atherosclerosis, Atherosclerosis, Mechanotransduction, Histone post-translational modifications, Cell signalling

## Abstract

Atherosclerotic lesions develop preferentially in arterial regions exposed to disturbed blood flow, where endothelial cells acquire an inflammatory phenotype. How disturbed flow induces endothelial cell inflammation is incompletely understood. Here we show that histone H3.3 phosphorylation at serine 31 (H3.3S31) regulates disturbed-flow-induced endothelial inflammation by allowing rapid induction of FOS and FOSB, required for inflammatory gene expression. We identified protein kinase N1 (PKN1) as the kinase responsible for disturbed-flow-induced H3.3S31 phosphorylation. Disturbed flow activates PKN1 in an integrin α5β1-dependent manner and induces its translocation into the nucleus, and PKN1 is also involved in the phosphorylation of the AP-1 transcription factor JUN. Mice with endothelium-specific PKN1 loss or endothelial expression of S31 phosphorylation-deficient H.3.3 mutants show reduced endothelial inflammation and disturbed-flow-induced vascular remodeling in vitro and in vivo. Together, we identified a pathway whereby disturbed flow through PKN1-mediated histone phosphorylation and FOS/FOSB induction promotes inflammatory gene expression and vascular inflammation.

## Abstract (web summary)

Jin et al. identified PKN1 as the kinase responsible for histone phosphorylation-mediated FOS/FOSB induction and vessel inflammation in regions of disturbed blood flow, highlighting an additional regulation layer for atherosclerotic plaque development.

## Main

Atherosclerosis is the major cause of myocardial infarction, ischemic stroke and peripheral artery disease and, thereby, contributes considerably to morbidity and mortality worldwide^1^. It is a chronic inflammatory disorder of large and medium-sized arteries. In addition to systemic risk factors, including obesity, diabetes mellitus, arterial hypertension, high plasma levels of low-density lipoprotein (LDL) cholesterol and triglycerides and others^2,3^, the local arterial microenvironment also has a strong effect on the development and progression of atherosclerotic lesions^4,5^. Atherosclerosis develops preferentially in arterial regions exposed to disturbed blood flow, such as vessel curvatures, bifurcations or branching points, whereas areas exposed to high laminar flow are resistant or are affected only at later stages of the disease^5–9^.

Different flow patterns are sensed by endothelial cells through various mechanosensitive proteins and protein complexes. Laminar and disturbed flow induce signal transduction processes in endothelial cells, which result in anti-atherogenic or pro-atherogenic phenotypes, respectively^7,10^. Disturbed flow, but not laminar flow, induces the expression of inflammatory genes encoding leukocyte adhesion molecules, including VCAM-1, ICAM- 1 or E-selectin, or chemokines, such as CCL2 (refs. ^11–13^). Inflammatory gene expression induced by disturbed flow is a complex process involving different signaling pathways and transcription factors and co-factors, including NF-κB, AP-1, EGR1 and YAP/TAZ. NF-κB, a well-established mediator of inflammatory gene expression in endothelial cells^11,12,14–16^, is activated by disturbed flow in a manner depending on integrins α5β1 and αvβ3 (refs. ^17–22^). Disturbed flow also promotes activation of the AP-1 transcription factors JUN and FOS^12,23–27^, and inflammatory gene expression in endothelial cells requires AP-1 activation^28–30^. However, the mechanisms underlying the activation of AP-1 by disturbed flow are not known.

The histone 3.3 (H3.3) variant is enriched in euchromatin and differs from the variants H3.1 and H3.2 in several residues, including amino acid 31, which is a serine in H3.3 and an alanine in the other variants^31^. Phosphorylation of serine 31 was recently shown to play an important role in gene regulation^32–34^. In murine embryonic stem cells (ESCs), H3.3 phosphorylation at serine 31 stimulates activity of the p300/CBP histone acetyl transferase, resulting in H3K27 acetylation at enhancer sequences required for ESC differentiation^32^. During activation of macrophages by lipopolysaccharide (LPS), H3.3 phosphorylation at serine 31 was shown to recruit the H3K36 methyl transferase SETD2, a component of the active transcription machinery, resulting in H3K36 trimethylation and rapid gene induction, including the dissociation of the transcription repressor ZMYND11 (ref. ^34^). Thus, phosphorylation of H3.3S31 appears to play an important role in rapid induction of gene expression programs. However, how H3.3S31 phosphorylation is regulated is poorly understood.

## Results

### Disturbed induced FOS/FOSB and endothelial inflammation

**Fig. 1:**
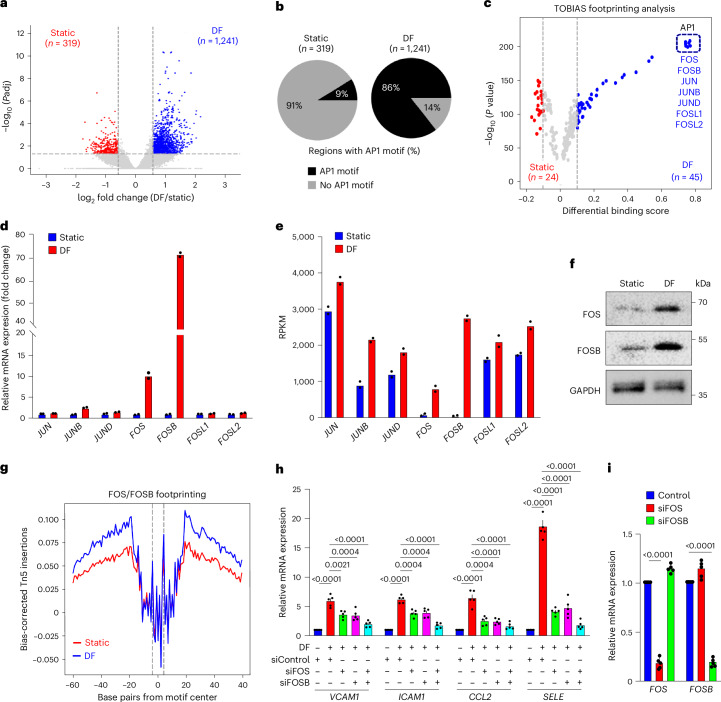
*FOS*/*FOSB* are induced by disturbed flow and mediate endothelial inflammation. **a**, Volcano plot comparing chromatin accessibility peaks in HUAECs kept under static conditions or exposed to disturbed flow (DF) for 3 h. **b**, Analysis of motif enrichment in genomic regions with increased accessibility in HUAECs reveals the considerable enrichment of AP-1 motifs. **c**, Volcano plot depicting results from TOBIAS. Red points depict motifs that are enriched and have a transcription factor (TF) footprint under static conditions; blue points depict motifs that are enriched and have a TF footprint when cells were exposed to DF. **d**–**f**, HUAECs were kept under static conditions or were exposed to DF for 3 h, and expression of the indicated AP-1 family members was assessed by RNA-seq (**d**,**e**) (*n* = 2) or immunoblotting (**f**) (*n* = 3 independent experiments). **g**, Aggregate ATAC-seq footprints of the FOS/FOSB motif in static and DF. Dashed lines represent the borders of the 11-bp FOS/FOSB motif. Curves represent normalized ATAC cut site signal summarized over all possible binding sites with a FOS/FOSB motif overlapped by a peak (*n* = 23,667). **h**,**i**, HUAECs were transfected with control siRNA (control) or siRNA directed against *FOS* or *FOSB* and were exposed to DF for 3 h. Expression of inflammatory genes (**h**) and *FOS*/*FOSB* (**i**) was analyzed by qRT–PCR (*n* = 5 independent experiments). Data are shown as mean ± s.e.m.; the *P* values are given in the figure (two-way ANOVA with Bonferroni’s post hoc test). bp, base pair; RPKM, reads per kilobase per million mapped reads. Source data

**Extended Data Fig. 1:**
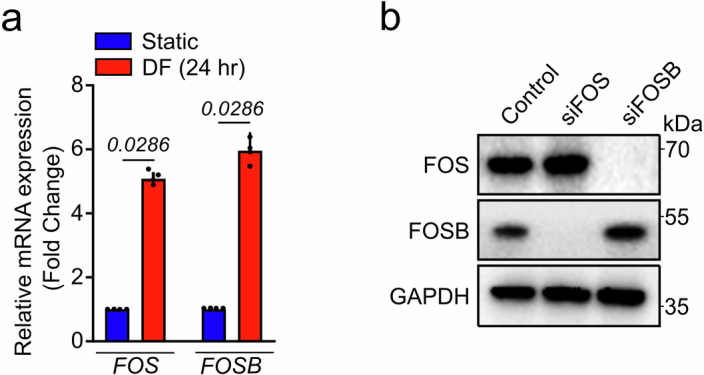
Disturbed flow-induced *FOS*/*FOSB* expression and FOS/FOSB knock-down efficiency. **a**, HUAECs were exposed to disturbed flow (4 dynes/cm^2^) for 24 hours, and *FOS* and *FOSB* levels were determined by qRT-PCR (*n* = 4 independent experiments). **b**, Validation of efficiency of siRNA-mediated knock-down of FOS and FOSB by immunoblotting using specific antibodies directed against FOS and FOSB. Analysis of GAPDH expression served as a loading control. (*n* = 3 independent experiments). Data are shown as mean ± s.e.m.; the p-values are given in the figure (Mann-Whitney two-sided test). Source data

To identify early changes in endothelial chromatin accessibility induced by disturbed flow, we performed the assay of transposase-accessible chromatin coupled to high-throughput sequencing (ATAC-seq) in human umbilical artery endothelial cells (HUAECs) exposed to disturbed flow. Comparison of chromatin accessibility between non-flowed cells and cells exposed to disturbed flow for 3 h revealed a large number of genomic regions that were more accessible in cells exposed to flow (Fig. 1a). To identify transcription factors that are recruited shortly after exposure of endothelial cells to disturbed flow and that may be involved in chromatin remodeling and transcriptional regulation, we performed transcription factor motif analysis in the 1,241 regions that were more accessible after flow exposure. The most highly enriched motifs included those that bind multiple members of the AP-1 transcription factor family. AP-1 motifs were found in 86% of the regions that became more accessible after flow compared to 9% under static conditions (Fig. 1b). Further bioinformatic analysis to predict transcription factor occupancy using footprint analysis (transcription factor occupancy prediction by investigating ATAC-seq signal (TOBIAS)) in the ATAC-seq dataset^35^ showed a considerable gain of putative transcription factor binding at AP-1 motifs in the accessible chromatin from HUAECs exposed to disturbed flow compared to cells kept under static conditions (Fig. 1c). When looking in more detail, we found that the AP-1 transcription factors *JUN*, *JU*N*B* and *JUND* were expressed at relatively high levels and that their expression was only slightly increased by disturbed flow (Fig. 1d,e). In contrast, *FOS* and *FOSB* were present at very low levels but showed strong induction shortly after exposure of cells to disturbed flow as well as after 24 h of disturbed flow (Fig. 1d,e and Extended Data Fig. 1a). The strong upregulation of FOS and FOSB in response to disturbed flow could also be seen on the protein level (Fig. 1f). Analysis of FOS/FOSB binding sites by footprint analysis showed a noticeable increase in endothelial cells exposed to disturbed flow when compared to cells kept under static conditions (Fig. 1g). Knockdown of FOS or FOSB alone partially inhibited disturbed-flow-induced expression of inflammatory genes, including *VCAM1*, *ICAM1*, *CCL2* and *SELE*, and knockdown of both FOS and FOSB blocked induction of inflammatory genes (Fig. 1h,i and Extended Data Fig. 1b). These data confirm older studies showing that AP-1 transcription factors are activated by disturbed flow^23–25,27^ and demonstrate that induction of *FOS/FOSB* expression is required for disturbed-flow-induced inflammatory gene expression.

### PKN1 mediates disturbed-flow-induced expression of *FOS*/*FOSB*

**Fig. 2:**
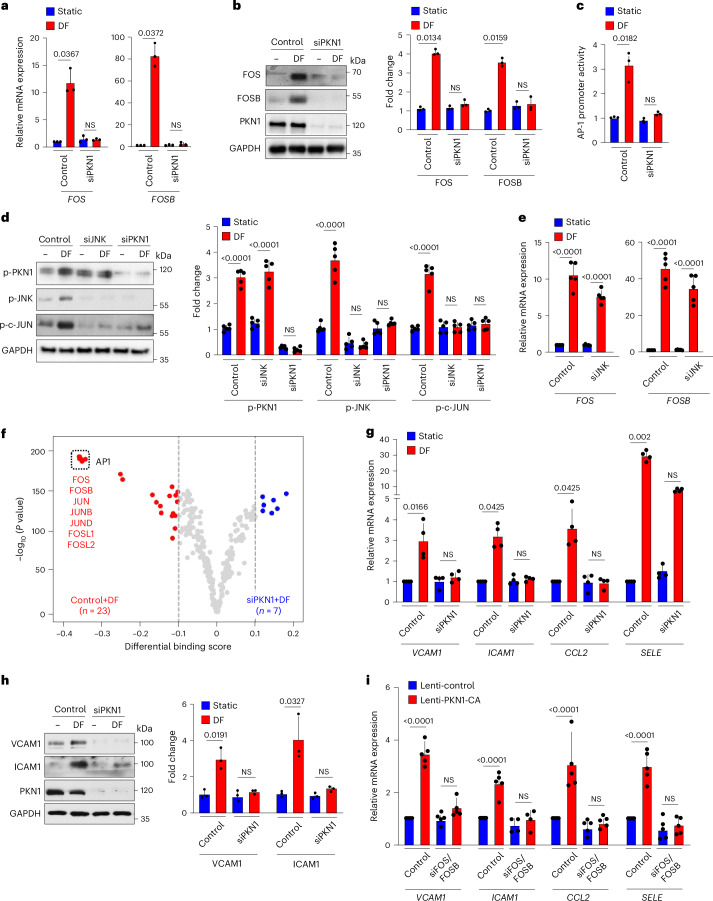
PKN1 mediates disturbed-flow-induced expression of *FOS*/*FOSB.* **a**–**c**, HUAECs were transfected with control siRNA or an siRNA against *PKN1* and were exposed to disturbed flow (DF) for 3 h as described above. FOS and FOSB levels were determined by qRT–PCR (**a**) or immunoblotting (**b**), and AP-1 promoter activity (**c**) was determined by luciferase reporter assay after transfection of cells with AP-1 promoter luciferase reporter construct (*n* = 3 independent experiments). **d**, HUAECs were transfected with control siRNA or siRNA against *JNK* or *PKN1* and were exposed to DF for 30 min. Phosphorylated PKN1, JNK or c-JUN was determined by immunoblotting (*n* = 5 independent experiments). **e**, HUAECs were transfected with control siRNA or an siRNA against *JNK* and were exposed to DF for 3 h. *FOS* and *FOSB* expression was determined by qRT–PCR (*n* = 5 independent experiments). **f**, Volcano plot depicting results from TOBIAS. Red points depict motifs that are enriched and have a transcription factor (TF) footprint in control cells exposed to disturbed flow; blue points depict motifs that are enriched and have a TF footprint in cells exposed DF after knockdown of PKN1. **g**,**h**, HUAECs were transfected with control siRNA or siRNA against *PKN1* and were exposed to DF for 3 h. Inflammatory gene expression was analyzed by qRT–PCR (**g**, *n* = 4 independent experiments) or immunoblotting (**h**, *n* = 3 independent experiments). **i**, The constitutively active mutant of PKN1 (PKN-CA) was expressed by lentiviral transduction in HUAECs, and expression of inflammatory genes was analyzed by qRT–PCR. Non-transducing lentivirus was used as a control (*n* = 5 independent experiments). Data are shown as mean ± s.e.m.; the *P* values are given in the figure (Kruskal–Wallis test (**a**–**c**,**g**,**h**) and one-way ANOVA with Tukey’s post hoc test (**d**,**e**,**i**)). NS, not significant. Source data

**Extended Data Fig. 2:**
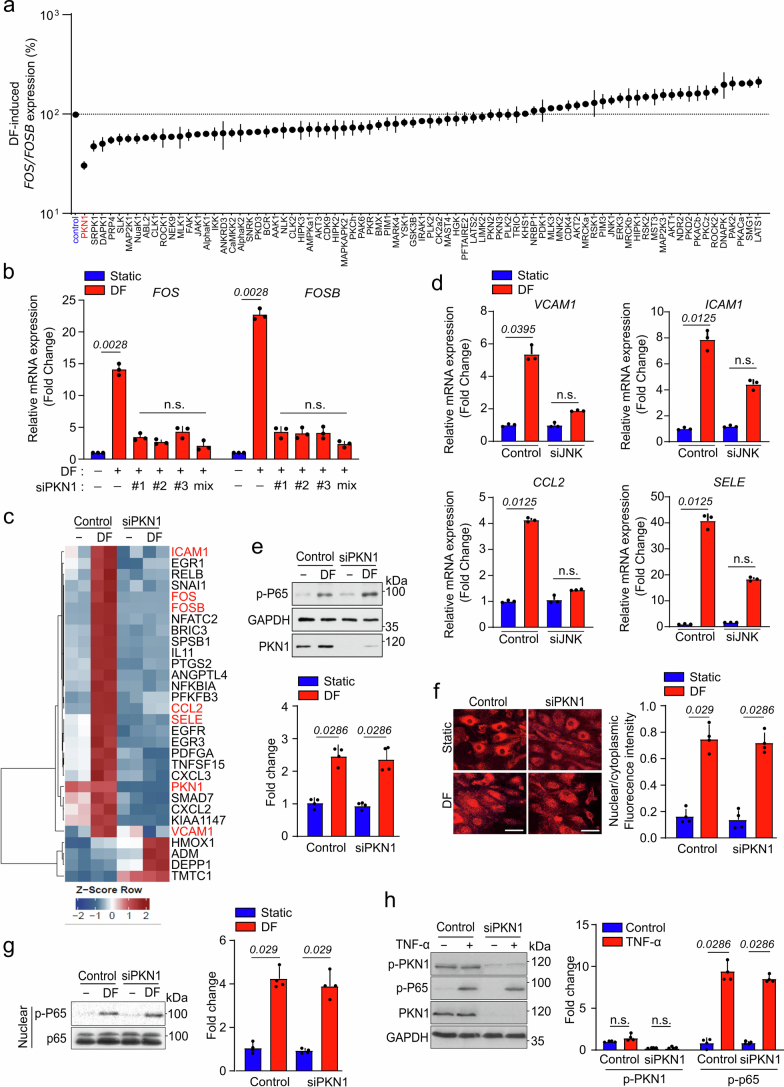
Effect of PKN1 knock-down in HUAECs on gene expression and NF-κB activation in response to disturbed flow and TNF. **a**, HUAECs were transfected with a control siRNA or siRNAs directed against different protein kinases and were exposed to disturbed flow (DF). Thereafter, flow-induced *FOS* and *FOSB* expression was determined by qRT-PCR. The plot shows the ranked average ratios of at least 3 independent experiments. **b**, HUAECs were transfected with control siRNA or different siRNAs against *PKN1* and were exposed to disturbed flow (DF) for 3 hrs. *FOS* and *FOSB* levels were determined by qRT-PCR (*n* = 3 independent experiments). **c**, HUAECs were transfected with control siRNA or siRNA against *PKN1* and were kept under static conditions (-) or were exposed to disturbed flow (DF) for 3 hours. The heatmap shows normalized expression of upregulated and downregulated genes. **d**, HUAECs were transfected with control siRNA or an siRNA against JNK and were exposed to disturbed flow (DF) for 3 hours. Thereafter, inflammatory gene expression was determined by qRT-PCR (*n* = 3 independent experiments). **e-h**, HUAECs were transfected with control siRNA or siRNA against *PKN1* and were exposed to disturbed flow (DF) for 30 min or were kept under static conditions (e-g) or were incubated in the absence or presence of 10 ng/ml TNF for 30 min (h). Thereafter, phosphorylated p65 or PKN1 were determined by immunoblotting (e, h), or nuclear p65 localization was determined by staining (f) or immunoblotting (g) (*n* = 4 independent experiments). Scale bar: 50 μm. Data are shown as mean ± s.e.m.; the p-values are given in the Fig. (2-way ANOVA with Bonferroni’s post hoc test (a), Kruskal-Wallis test (b, d-h)). Source data

In an attempt to identify signaling mechanisms involved in disturbed-flow-induced induction of *FOS* and *FOSB* expression, we performed an siRNA-mediated knockdown of 80 protein kinases highly expressed in HUAECs and determined the effect on induction of both AP-1 transcription factors induced by disturbed flow (Extended Data Fig. 2a). PKN1 turned out to be the kinase whose knockdown most strongly reduced disturbed-flow-induced *FOS/FOSB* induction. Alternative siRNAs directed against PKN1 also blocked disturbed-flow-induced expression of FOS/FOSB both on the RNA as well as on the protein level and prevented disturbed-flow-induced AP-1 promoter activation (Fig. 2a–c and Extended Data Fig. 2b). When we analyzed disturbed-flow-induced JUN phosphorylation, we found that this effect was blocked after knockdown of Jun N-terminal kinase (JNK) as well as after knockdown of PKN1 (Fig. 2d), and knockdown of PKN1 also abrogated disturbed-flow-induced JNK phosphorylation (Fig. 2d). However, knockdown of JNK did not affect disturbed-flow-induced induction of *FOS* and *FOSB* expression (Fig. 2e). This indicates that PKN1 is regulating not only the expression of *FOS* and *FOSB* but also the phosphorylation of JUN through the activation of JNK. The critical role of PKN1 in disturbed flow-induced induction and activation of AP-1 transcription factors was also obvious when we compared transcription factor binding between HUAECs exposed to disturbed flow in control cells and cells after knockdown of PKN1 (Fig. 2f). These data showed a strongly reduced number of AP-1 transcription factor motifs in accessible regions after knockdown of PKN1 (Fig. 2f).

Consistent with a critical role of AP-1 in disturbed-flow-induced expression of inflammatory genes, we found that suppression of PKN1 expression blocked or inhibited induction of inflammatory genes by disturbed flow on the RNA level as well as on the protein level (Fig. 2g,h and Extended Data Fig. 2c), an effect also seen after knockdown of JNK (Extended Data Fig. 2d). In addition, a constitutively active version of PKN1 expressed in HUAECs increased inflammatory gene expression, and the effect was lost after knockdown of FOS/FOSB (Fig. 2i). Knockdown of PKN1 had no effect on phosphorylation or nuclear translocation of the NF-κB component p65 induced by disturbed flow or tumor necrosis factor (TNF) (Extended Data Fig. 2e–h). These data indicate that PKN1 specifically mediates disturbed-flow-induced phosphorylation of JUN and expression of *FOS* and *FOSB*, which are required for disturbed-flow-induced expression of inflammatory genes.

### Disturbed flow induces PKN1 activation via integrin α5β1

**Fig. 3:**
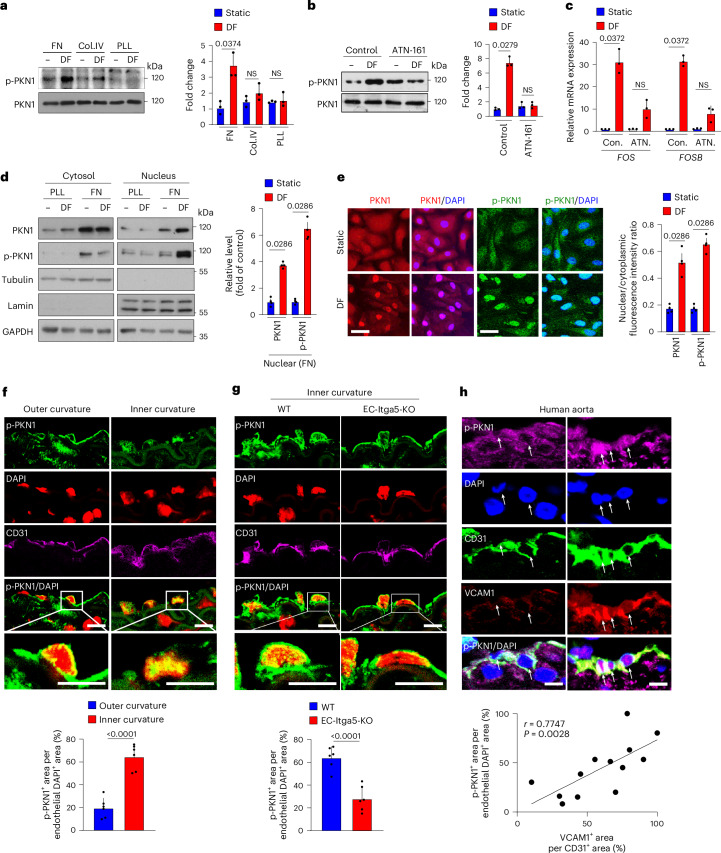
Disturbed-flow-induced PKN1 activation and nuclear translocation is mediated by integrin α5β1. **a**, HUAECs were plated on fibronectin (FN), collagen IV (Col.IV) or poly-l-lysine (PLL) and were kept under static conditions (−) or were exposed to disturbed flow (DF) for 60 min. Total and phosphorylated PKN1 was determined by immunoblotting (*n* = 3 independent experiments). **b**,**c**, HUAECs were exposed to DF for 30 min (**b**) or 3 h (**c**) or were kept under static conditions after pre-treatment for 30 min without or with the integrin α5β1 antagonist ATN-161 (10 μM). Total and phosphorylated PKN1 was determined by immunoblotting (**b**), and *FOS*/*FOSB* expression was analyzed by qRT–PCR (**c**) (*n* = 3 independent experiments). **d**, HUAECs on FN or PLL were exposed to static conditions or DF for 1 h, after which total and phosphorylated PKN1 distribution in the nucleus and cytoplasm was analyzed by immunoblotting (*n* = 3 independent experiments). **e**, HUAECs were exposed to DF for 1 h or kept under static conditions and then stained with antibodies against PKN1 (red) and p-PKN1 (green), along with DAPI (blue). The ratio of nuclear and cytoplasmic PKN1 or p-PKN1 was quantified (*n* = 3 independent experiments). Scale bar, 20 μm. **f**,**g**, Cross-sections of the inner (**f**,**g**) and outer (**f**) curvatures of aortic arches from wild-type (WT) (**f**,**g**) or EC-Itga5-KO (**g**) mice were stained with antibodies against phosphorylated PKN1 (green) and CD31 (purple) as well as with DAPI (red). The bar diagrams show percentage of phosphorylated PKN1-positive area per endothelial DAPI-positive area. Scale bar, 20 μm (*n* = 6 mice per group; at least three sections were analyzed per animal). **h**, Representative immunofluorescence confocal images of sections of the human aorta. Sections were stained with antibodies against phosphorylated PKN1 (purple), CD31 (green) and VCAM1 (red) as well as with DAPI (blue). Arrows indicate endothelial marker-positive cells. The Spearman correlation analysis showed a significant relationship (*r* = 0.7747, *P* = 0.0028) (*n* = 13 different patients; at least three sections per patient sample were analyzed). Scale bar, 20 µm. Data are shown as mean ± s.e.m.; the *P* values are given in the figure (Kruskal–Wallis test (**a**–**c**), Mann–Whitney two-sided test (**d**,**e**) or unpaired two-sided *t*-test (**f**,**g**)). NS, not significant; Con., control; ATN., ATN-161. Source data

**Extended Data Fig. 3:**
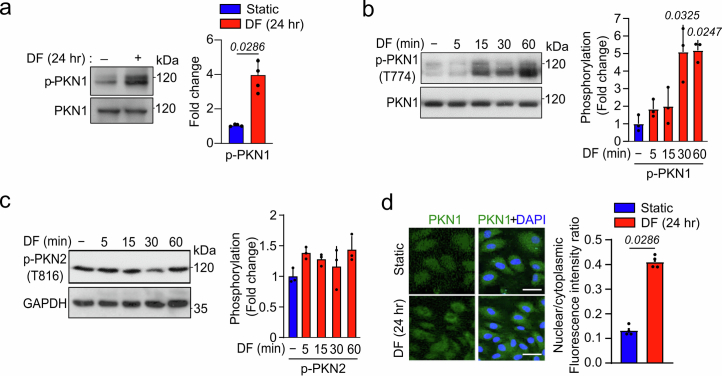
Activation of PKN1 by disturbed flow. **a**, HUAECs were exposed to disturbed flow (4 dynes/cm^2^) for 24 hours. PKN1 phosphorylation was determined by immunoblotting using an antibody directed against phosphorylated PKN1. The bar diagram shows the statistical analysis (*n* = 4 independent experiments). **b**,**c**, HUAECs were exposed to disturbed flow for the indicated time periods. Thereafter, phosphorylation of PKN1 (b) or PKN2 (c) was determined by immunoblotting using phosphosite-specific antibodies (*n* = 3 independent experiments). **d**, HUAECs were exposed to disturbed flow (4 dynes/cm^2^) for 24 hours. PKN1 localization was determined by immunofluorescence staining using an antibody directed against PKN1 (green) and with DAPI (blue). The bar diagram shows the statistical analysis (*n* = 4 independent experiments). Data are shown as mean ± s.e.m.; the p-values are given in the figure (Mann-Whitney two-sided test (a, d), Kruskal-Wallis test (b, c)). Source data

After applying disturbed flow to endothelial cells, we detected phosphorylation of PKN1 at threonine 774 (Fig. 3a and Extended Data Fig. 3a), which was shown to result in PKN1 activation^36^. Disturbed-flow-induced PKN1 phosphorylation occurred in a time-dependent manner and was specific as PKN2 was not phosphorylated in response to disturbed flow (Extended Data Fig. 3b,c). Disturbed-flow-induced PKN1 activation was not seen in cells cultured on plates coated with poly-l-lysine or collagen but depended on fibronectin coating of plates (Fig. 3a). As shown in Fig. 3b,c, blockade of the fibronectin-binding integrin α5β1 by ATN-161 inhibited disturbed-flow-induced phosphorylation of PKN1 as well as induction of *FOS* and *FOSB* expression. This indicates an involvement of integrin α5β1, which was shown to mediate disturbed-flow-induced inflammatory signaling and inflammatory gene expression^17,18,37,38^. When studying the effect of disturbed flow on the cellular localization of PKN1, we noticed that disturbed flow induced strong nuclear translocation of total and phosphorylated PKN1 (Fig. 3d,e and Extended Data Fig. 3d), an effect seen only in cells grown on plates coated with fibronectin but not with poly-l-lysine (Fig. 3d). Consistent with a role of PKN1 in disturbed-flow-induced endothelial inflammation, we found an increased fraction of phosphorylated PKN1 in the nucleus of endothelial cells of the inner curvature of the aortic arch, which is mainly exposed to disturbed flow, compared to the outer curvature, which is exposed to laminar flow (Fig. 3f). In endothelium-specific integrin α5-deficient mice (Cdh5-CreERT2;*Itga5*^flox/flox^, hereafter referred to as EC-Itga5-KO), the nuclear localization of phosphorylated PKN1 in endothelial cells of the inner curvature was reduced compared to wild-type mice (Fig. 3g). We then studied nuclear localization of phosphorylated PKN1 in the human aorta, in which we compared areas without and with endothelial VCAM1 expression as an indicator of endothelial inflammation. As shown in Fig. 3h, endothelial expression of VCAM1 correlated with nuclear localization of phosphorylated PKN1. These data indicate that disturbed flow induces activation and nuclear translocation of PKN1 and that this is mediated by integrin α5β1.

### PKN1 phosphorylates H3.3S31 to promote *FOS*/*FOSB* expression

**Fig. 4:**
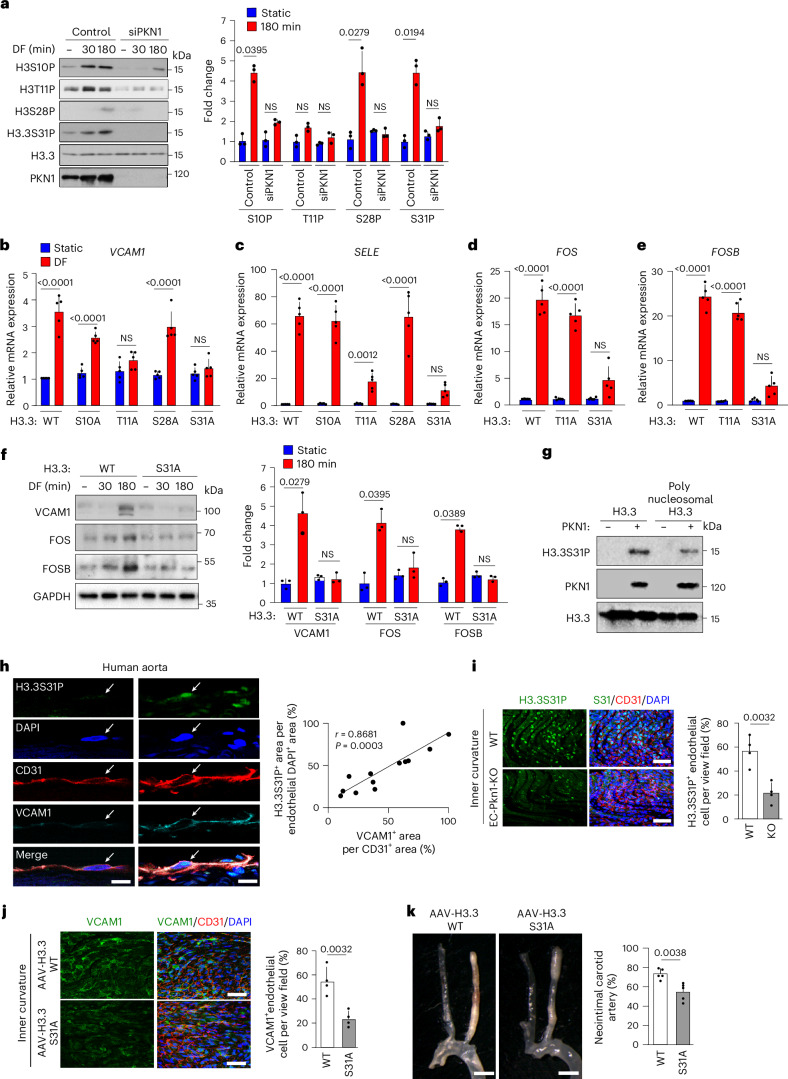
PKN1 phosphorylates histone H3.3 at serine 31 to promote *FOS*/*FOSB* expression and endothelial inflammation. **a**, HUAECs transfected with control siRNA or PKN1 siRNA were exposed to disturbed flow for specified time periods, and histone H3 phosphorylation was assessed by immunoblotting (*n* = 3 independent experiments). The presented immunoblots are from the same experiment as blots shown in Fig. 5a and include the same H3.3 loading control. **b**–**f**, Wild-type (WT) human H3.3 or mutants were expressed in HUAECs via lentiviral transduction after siRNA-mediated H3.3 knockdown; cells were subjected to static conditions or disturbed flow for 3 h; and mRNA levels of *VCAM1* (**b**). SELE (**c**), *FOS* (**d**) and *FOSB* (**e**) were analyzed by qRT–PCR (*n* = 5 independent experiments) or protein levels were determined by immunoblotting (**f**, *n* = 3 independent experiments). **g**, Recombinant histone H3.3 or polynucleosomes were incubated with or without recombinant PKN1 and ATP and analyzed by immunoblotting for H3.3S31P, PKN1 and H3.3 (*n* = 3 independent experiments). **h**, Representative immunofluorescence confocal images of human aorta stained for phosphorylated H3.3S31 (green), CD31 (red), VCAM1 (cyan) and DAPI (blue). Arrows indicate endothelial marker (CD31)-positive cells. Shown is the Spearman correlation analysis (*n* = 13 different patients, at least three sections per patient). **i**, Representative en face immuno-confocal microscopy images of the aortic inner curvature from WT and EC-Pkn1-KO mice (*n* = 4 per group) stained with anti-H3.3S31P or anti-CD31 antibodies and DAPI. Immunofluorescence was quantified as the percentage of H3.3S31P-positive cells among CD31-positive cells per view field (*n* = 4 mice, at least three areas per mouse). **j**,**k**, Atheroprone *Ldlr*^−/−^ mice were infected with AAV2-QuadYF virus to transduce WT H3.3 or the phosphosite mutant H3.3S31A. En face images of the aortic inner curvature were obtained after VCAM1, CD31 and DAPI staining (**j**). Immunofluorescence was quantified as the percentage of VCAM1-positive cells among CD31-positive cells per field (**j**, *n* = 5 mice per group, at least three areas per mouse). Two weeks after carotid artery ligation, mice were euthanized, and light microscopy images of carotid arteries were analyzed for the percentage of neointima between the aorta and the ligation site (**k**, *n* = 6 mice per group). Bar lengths, 20 µm (**h**), 50 µm (**i**,**j**) and 5 mm (**k**). Data are shown as mean ± s.e.m.; the *P* values are given in the figure (Kruskal–Wallis test (**a**,**d**–**f**), one-way ANOVA with Tukey’s post hoc test (**b**,**c**), Mann–Whitney two-sided test (**i**,**j**) or unpaired two-sided *t-*test (**k**)). NS, not significant. Source data

**Extended Data Fig. 4:**
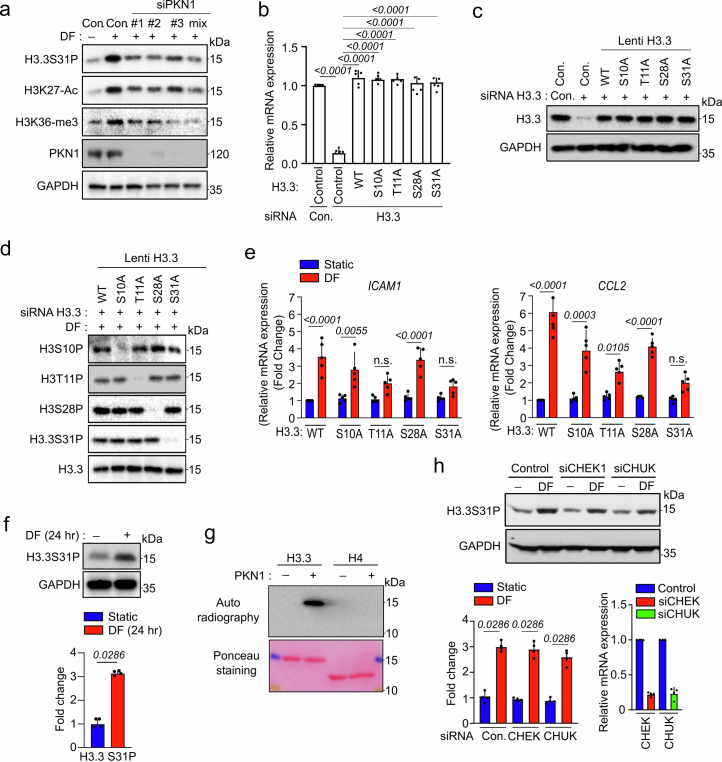
Role of histone H3.3 in endothelial cells and PKN1-mediated H3.3 phosphorylation. **a**, HUAECs were transfected with control siRNA or different siRNA against *PKN1* and were exposed to disturbed flow (DF) for 30 min. Histone H3.3 phosphorylation, H3K27 acetylation or H3K36 trimethylation was determined by immunoblotting. **b**-**d**, HUAECs were transfected with control siRNA (Con.) or siRNA directed against histone H3.3, and cells were infected with control (non-transducing) lentivirus (Con.) or lentivirus transducing wild-type (WT) H3.3 or the indicated mutants of H3.3. Shown is the relative H3.3 mRNA expression (b), (*n* = *5* independent experiments), the protein level determined by immunoblotting using an antibody directed against histone H3.3 (c) (*n* = 3 independent experiments) and the analysis of phosphorylation of histone H3 at the indicated phosphorylation sites using phosphosite-specific antibodies after exposure of HUAECs to disturbed flow for 60 min (d) (*n* = 3 independent experiments). **e**, Wild-type (WT) human H3.3 or the indicated mutants of H3.3 were expressed by lentiviral transduction after siRNA-mediated H3.3 knock-down in HUAECs, and cells were kept under static conditions or were exposed to disturbed flow (DF) for 3 h. Thereafter, *ICAM1* and *CCL2* expression was determined by qRT-PCR (*n* = 5 independent experiments). **f**, HUAECs were exposed to disturbed flow (4 dynes/cm^2^) for 24 hours, and phosphorylation of histone H3.3 at serine 31 was determined by immunoblotting (*n* = 4 independent experiments). The control immunoblot for GAPDH is from the same experiment as the one shown in Extended Data Fig. 6a. **g**, Recombinant histone H3.3 or H4 were incubated in kinase buffer without or with recombinant PKN1 and 50 µM [γ-^32^P]-ATP for 30 min at 30 °C. Thereafter, samples were separated by SDS-PAGE and analyzed by autoradiography and Ponceau staining. **h**, HUAECs were transfected with control siRNA or siRNA directed against RNAs encoding the kinases CHEK or IKKα (*CHEK1* or *CHUK*, respectively). Thereafter, cells were kept under static conditions (-) or were exposed to disturbed flow (DF) for 60 min. Phosphorylation level of H3.3S31 in lysates was determined by immunoblotting (*n* = 3 independent experiments). The bar diagrams show the statistical evaluation as well as the knock-down efficiency. Data are shown as mean ± s.e.m.; the p-values are given in the Fig. (1-way ANOVA with Tukey’s post hoc test (b, e), Mann-Whitney two-sided test (f), Kruskal-Wallis test (h)). Source data

Because PKN1 translocated to the nucleus in response to disturbed flow and because PKN1 was shown previously to phosphorylate histone H3 at threonine 11 to regulate transcriptional activity^39^, we tested whether disturbed flow induced phosphorylation of histone H3. We found that, within 30 min of disturbed flow, histone H3 became phosphorylated at serine 10 but not at threonine 11, which showed a non-significant tendency toward increased phosphorylation (Fig. 4a). In addition, a small phosphorylation after a longer time period could also be seen at serine 28 in response to disturbed flow (Fig. 4a). Cells express not only histone H3.1/2 but also the histone H3.3 variant, which, in addition, can be phosphorylated at serine 31 (ref. ^40^). We, therefore, tested the effect of disturbed flow on histone H3.3 serine 31 phosphorylation and found that it also increased within 30 min after exposure of cells to disturbed flow (Fig. 4a). Suppression of PKN1 expression resulted in loss of disturbed-flow-induced phosphorylation at each of the sites (Fig. 4a and Extended Data Fig. 4a). To test whether any of the PKN1-mediated histone H3.3 phosphorylation events were relevant for disturbed-flow-induced inflammatory gene expression, we suppressed expression of endogenous histone H3.3 using siRNA and infected HUAECs with lentivirus transducing wild-type H3.3 or the phosphosite mutants of H3.3 (Extended Data Fig. 4b–d). Although expression of H3.3(S10A) and H3.3(S28A) had no effect on disturbed-flow-induced inflammatory gene expression, expression of the phosphosite mutants H3.3(T11A) and H3.3(S31A) inhibited disturbed-flow-induced *VCAM1* and *SELE* as well as *ICAM1* and *CCL2* expression (Fig. 4b,c and Extended Data Fig. 4e). When testing the effect of the T11A and S31A mutants of H3.3 on disturbed-flow-induced expression of *FOS* and *FOSB*, we saw that only the H3.3(S31A) mutant had an effect (Fig. 4d,e), suggesting that histone H3 phosphorylation at threonine 11 is required for efficient induction of inflammatory gene expression through a FOS/FOSB-independent mechanism. Loss of flow-induced expression of VCAM1 and FOS/FOSB after expression of the H3.3 phosphosite mutant H3.3(S31A) was also seen on the protein level (Fig. 4f). We, therefore, focused on the PKN1-mediated regulation of H3.3 serine 31 phosphorylation, which could be observed even 24 h after exposure to disturbed flow (Extended Data Fig. 4f).

To determine whether PKN1 can directly phosphorylate histone H3.3 at serine 31, we incubated recombinant histone H3.3 alone or as part of a recombinant polynucleosome together with recombinant PKN1 in the presence of ATP and observed PKN1-dependent phosphorylation of serine 31 (Fig. 4g). In contrast to recombinant histone H3.3, recombinant histone H4 was not phosphorylated by PKN1, indicating that PKN1-dependent phosphorylation occurred with some specificity (Extended Data Fig. 4g). These data provide evidence that PKN1 can directly phosphorylate histone H3.3 at serine residue 31. Knockdown of IKKα (CHUK) or CHEK1, which were shown to be able to phosphorylate H3.3 at serine 31 (refs. ^41,42^), had no effect on H3.3S31 phosphorylation in response to disturbed flow (Extended Data Fig. 4h).

**Extended Data Fig. 5:**
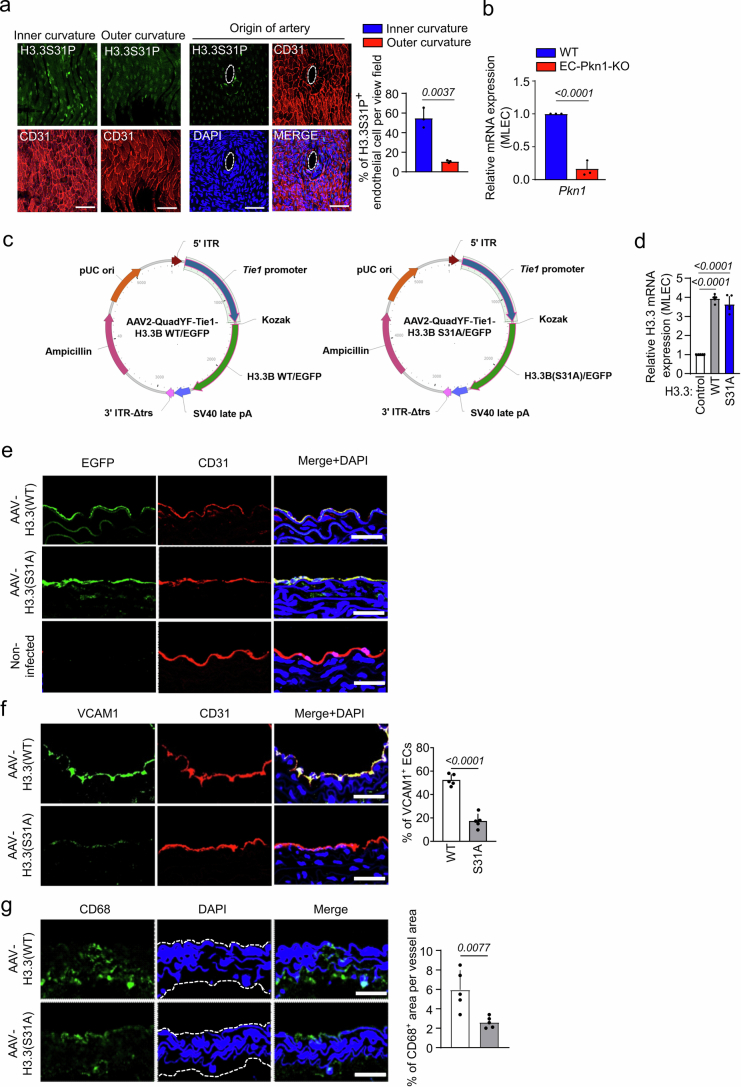
Endothelial histone H3.3 phosphorylation *in vivo* and AAV2-mediated expression of histone H3.3. **a**, Shown are representative en face immuno-confocal microscopy images of the inner and outer curvature of the aortic arch (left panels), or of the origin of an intercostal artery of the descending aorta (right panels) from wild-type mice stained with antibodies directed against H3.3S31P and CD31 as well as with DAPI. Immunofluorescence staining was quantified as the percentage of phosphorylated H3.3S31-positive cells among CD31-positive cells per view field (*n* = 3 mice per group; at least 3 areas were analyzed per animal). **b**, *Pkn1* expression in mouse lung endothelial cells (MLECs) from wild-type (WT) and EC-Pkn1-KO mice (*n* = 3 mice per group). **c**, Map of vectors used to generate AAV2 particles transducing wild-type (WT) and mutant H3.3. Histone 3.3B(WT) or the H3.3(S31A) mutant are C-terminally fused with EGFP, and their expression is driven by the *Tie1* promoter. The constructs were used to generate quadruplet mutant AAV2 viral particles referred to as AAV2-QuadYF. **d**, Relative histone H3.3 mRNA expression in mouse lung endothelial cells (MLECs) prepared from *Ldlr*^-/-^ mice, which had been infected with empty, non-transducing AAV2-QuandYF virus (control) or AAV2-QuandYF-transducing wild-type H3.3 (WT) or the phospho-site mutant H3.3S31A (*n* = 5 different mice). **e**, Images of carotid artery sections from AAV2-QuadYF-infected or non-infected mice stained for EGFP (green), CD31 (red) and with DAPI (blue) (shown is a representative of 3 independently performed experiments). **f**, **g**, Atheroprone *Ldlr*^−/−^ mice were infected with AAV2-QuadYF virus transducing wild-type (WT) H3.3 or the phosphosite mutant H3.3(S31A). Cross sections of the left common carotid artery (ligated artery) were stained with antibodies against VCAM1 (f) or CD68 (g) and against CD31 (f) as well as with DAPI (blue). Immunofluorescence staining was quantified (*n* = 5 mice per group; at least 3 areas per animal were analyzed). The boundary of the vessel area in (g) is indicated by a dashed line. Scale bars: 50 μm (a), 20 μm (e-g). Data are shown as mean ± s.e.m.; the p-values are given in the figure (Mann-Whitney two-sided test (a, b), unpaired two-sided *t* test (d, f, g)). Source data

Consistent with a role of PKN1-mediated H3.3S31 phosphorylation in endothelial inflammation, we found that the number of endothelial cells showing phosphorylated H3.3S31 was higher in areas exposed to disturbed flow, such as the inner curvature of the aortic arch or vascular branching points, compared to areas exposed to laminar flow, such as the outer curvature of the aortic arch (Extended Data Fig. 5a). Increased H3.3S31 phosphorylation was also seen in the human aorta in areas with increased VCAM1 expression (Fig. 4h). Phosphorylation of endothelial H3.3S31 was, however, reduced in the inner curvature of endothelium-specific PKN1-deficient mice (Tek-CreER^T2^;*Pkn1*^flox/flox^, hereafter referred to EC-Pkn1-KO) (Fig. 4i and Extended Data Fig. 5b). Expression of H3.3(S31A) in endothelial cells in vivo using adeno-associated virus 2 (AAV2) (Extended Data Fig. 5c–e) led to a reduced *Vcam1* expression in endothelial cells of the inner curvature of the aortic arch (Fig. 4j) and reduced inflammation and neointima area after partial carotid artery ligation—a model for acute disturbed-flow-induced endothelial dysfunction and atherosclerosis (Fig. 4k and Extended Data Fig. 5f,g).

### Induction of *FOS/FOSB* by PKN1 involves EP300 and SETD2

**Fig. 5:**
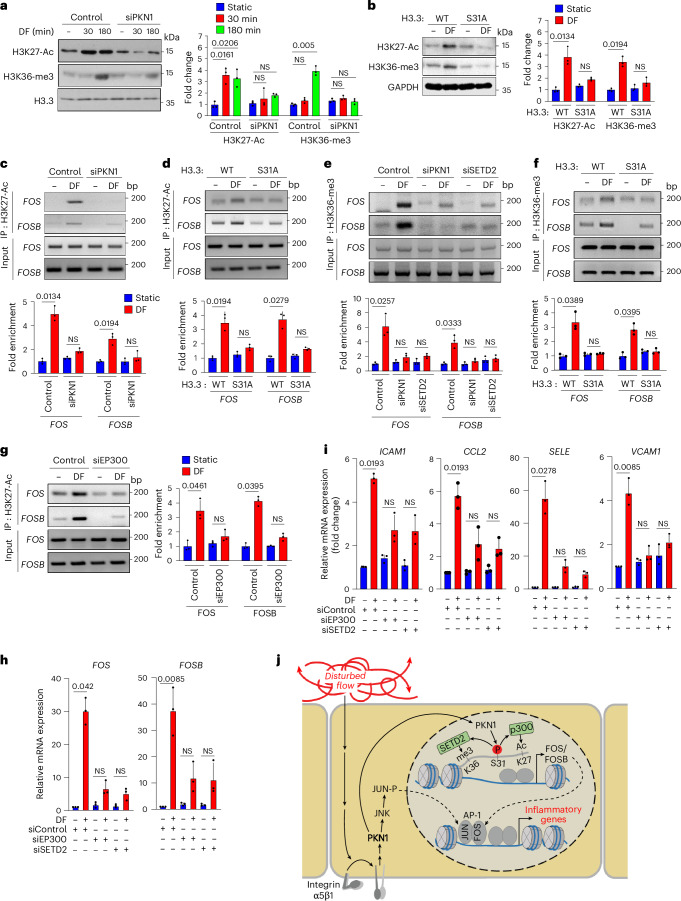
PKN1-induced *FOS*/*FOSB* induction through phosphorylation of H3.3S31 involves EP300 and SETD2. **a**, HUAECs were transfected with a control siRNA or siRNAs directed against *PKN1*. Thereafter, cells were exposed to disturbed flow (DF) for the indicated time periods. H3K27 acetylation or H3K36 tri-methylation in lysates was determined by immunoblotting (*n* = 3 independent experiments). The presented immunoblots are from the same experiment as the blots shown in Fig. 4a and include the same H3.3 loading control. **b**, Wild-type (WT) or mutant (S31A) human H3.3 was expressed by lentiviral transduction after siRNA-mediated H3.3 knockdown in HUAECs. Thereafter, cells were exposed to DF for 3 h or were kept under static conditions. H3K27 acetylation or H3K36 tri-methylation in lysates was determined by immunoblotting (*n* = 3 independent experiments). **c**–**g**, HUAECs were transfected with a control siRNA or siRNAs directed against *PKN1* (**c**,**e**), *SETD2* (**e**) or *EP300* (**g**) and were then exposed to DF for 90 min. Alternatively, wild-type (WT) or mutant (S31A) human H3.3 was expressed by lentiviral transduction after siRNA-mediated knockdown of H3.3 (**d**,**f**) in HUAECs. ChIP assay was performed to detect the enrichment of H3K27 acetylation (**c**,**d**,**g**) or H3K36 tri-methylation (**e**,**f**) in the *FOS*/*FOSB* promoter (**c**,**d**,**g**) or gene body (**e**,**f**) (*n* = 3 independent experiments). **h**,**i**, HUAECs were transfected with control siRNA or siRNAs directed against *EP300* or *SETD2* and were exposed to DF for 3 h. DF-induced expression of *FOS* and *FOSB* (**h**) or inflammatory genes (**i**) was analyzed by qRT–PCR (*n* = 3 independent experiments). **j**, Schematic representation of the role of PKN1 in mediating DF-induced inflammatory gene expression in endothelial cells. Data are shown as mean ± s.e.m.; the *P* values are given in the figure (Kruskal–Wallis test (**a**–**i**)). NS, not significant. Source data

**Extended Data Fig. 6:**
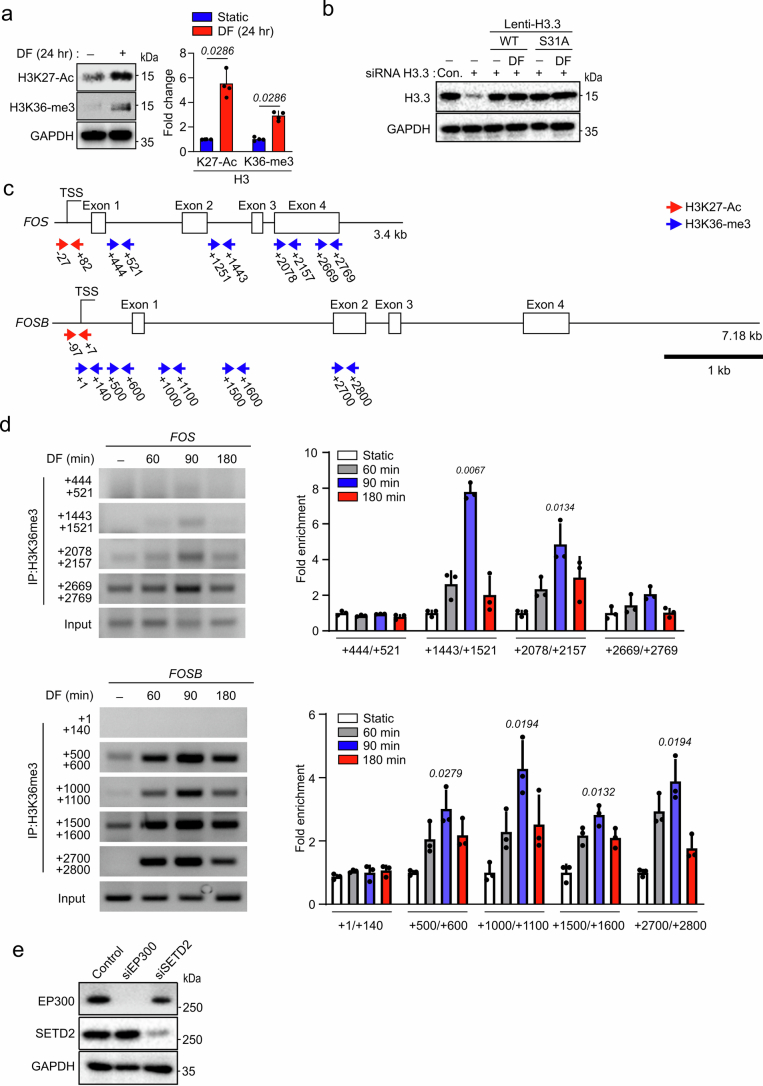
Disturbed flow induced endothelial histone modification. **a**, HUAECs were exposed to disturbed flow (4 dynes/cm^2^) for 24 hours, and H3K27 acetylation or H3K36 trimethylation in lysates were determined by immunoblotting (*n* = 4 independent experiments). The control immunoblot for GAPDH is from the same experiment as the one shown in Extended Data Fig. 4f. **b**, Wild-type (WT) H3.3 or the H3.3S31A mutant were expressed by lentiviral transduction following siRNA-mediated H3.3 knockdown in HUAECs. Thereafter, HUAECs were kept under static conditions (-) or were exposed to disturbed flow for 30 min, and the expression of histone H3.3 was analyzed by immunoblotting. **c**, Schematic representation of the human *FOS* and *FOSB* genes with the position of primers (arrows) used for ChIP assay. TSS, transcription start site. **d**, To study disturbed flow-induced enrichment of H3K36me3 on different parts of the *FOS* and *FOSB* gene bodies, HUAECs were exposed to disturbed flow for the indicated time periods followed by ChIP assay with an anti-H3K36me3 antibody. Thereafter, PCR was performed with the indicated primer pairs. Primer pairs amplifying the regions from +2078 to 2157 of the *FOS* gene and from +2700 to 2800 of the *FOSB* gene were used for subsequent experiments. Bar diagrams show the quantification and statistical analysis (*n* = 3 independent experiments). **e**, Validation of efficiency of siRNA-mediated knock-down of *EP300* and *SETD2* in HUAECs by immunoblotting using specific antibodies directed against EP300 and SETD2. (*n* = 3 independent experiments). Data are shown as mean ± s.e.m.; the p-values are given in the figure (Mann-Whitney two-sided test (a), Kruskal-Wallis test (d)). Source data

H3.3 serine 31 phosphorylation was shown to promote gene transcription by stimulating SETD2-dependent tri-methylation of H3K36 as well as by enhancing the enzymatic activity of the histone acetyl transferase p300 (EP300) to promote acetylation of H3K27 (refs. ^32,34^). Whereas disturbed flow increased acetylation of H3K27 within 30 min of disturbed flow, tri-methylation of H3K36 was not seen after 30 min of disturbed flow but strongly increased within 3 h of disturbed flow (Fig. 5a), and both effects were still observed after 24 h of exposure to disturbed flow (Extended Data Fig. 6a). Disturbed-flow-induced H3K27 acetylation and H3K36 tri-methylation were blocked by knockdown of PKN1 (Fig. 5a) as well as by expression of the S31A phosphosite mutant of H3.3 (Fig. 5b and Extended Data Fig. 6b). Using chromatin immunoprecipitation (ChIP), we analyzed the effect of a knockdown of PKN1 or expression of the H3.3(S31A) mutant on disturbed-flow-dependent presence of H3K27 acetylation in the promoter regions of the *FOS* and *FOSB* genes^43,44^ as well as on flow-dependent association of tri-methylated H3K36 with the *FOS* and *FOSB* gene bodies^45^ (Extended Data Fig. 6c,d). When ChIP assays were performed with an anti-H3K27ac or an anti-H3K36-me3 antibody, enrichment of H3K27 acetylation and H3K36 tri-methylation, respectively, could be observed on the respective regions of the *FOS* and *FOSB* genes (Fig. 5c–g). This disturbed-flow-induced effect was not seen after knockdown of PKN1 (Fig. 5c,e) or after expression of the S31A mutant of H3.3 (Fig. 5d,f). Similarly, knockdown of EP300 or SETD2 blocked flow-induced H3K27 acetylation associated with the *FOS/FOSB* promoter region and tri-methylation of H3K36 associated with the *FOS/FOSB* gene body, respectively (Fig. 5e,g and Extended Data Fig. 6e). This strongly suggests that PKN1-mediated phosphorylation of H3.3S31 in response to disturbed flow leads to increased *FOS/FOSB* expression by activation of the enzymatic activity of p300 and subsequent H3K27 acetylation as well as by SETD2-mediated H3K36 tri-methylation. This is further supported by the observation that knockdown of both EP300 and SETD2 inhibited flow-induced expression of *FOS*/*FOSB* and inflammatory genes (Fig. 5h–j).

### PKN1 mediates endothelial inflammation and atherosclerosis

**Fig. 6:**
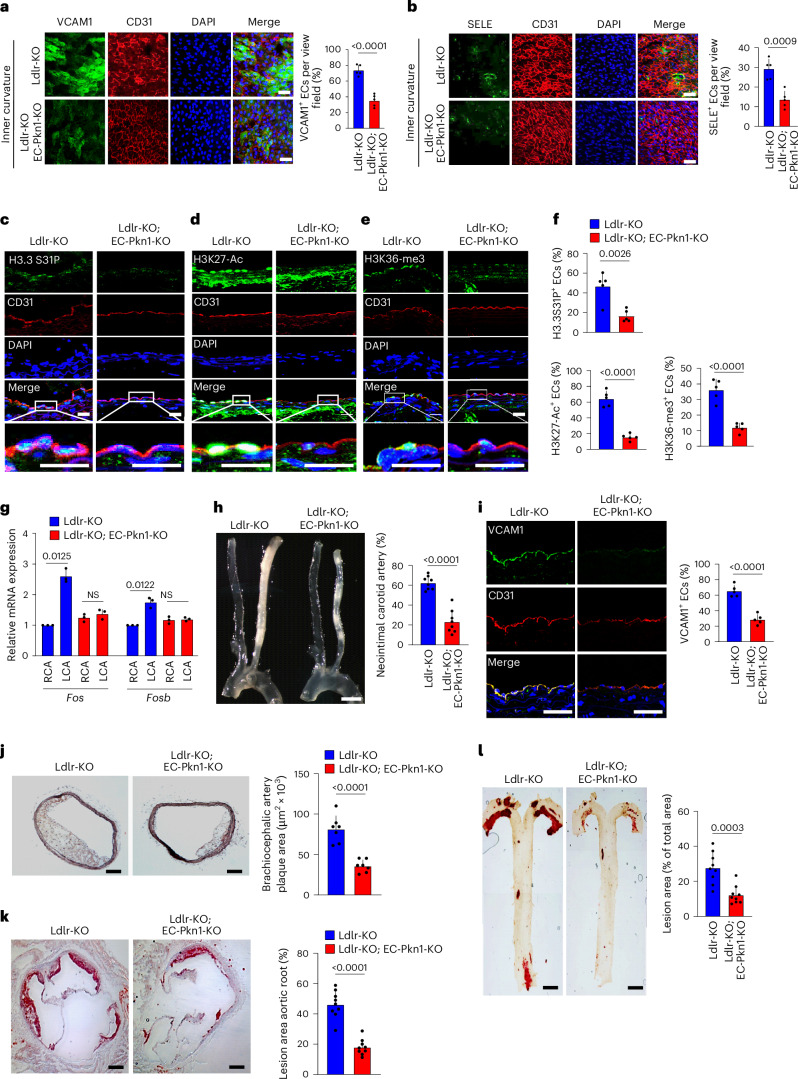
PKN1 mediates endothelial inflammation and progression of atherosclerosis in vivo*.* **a**,**b**, Shown are representative en face immuno-confocal microscopy images of the inner curvature from 12-week-old atherosclerosis-prone *Ldlr*^−/−^ mice without (Ldlr-KO) or with endothelium-specific Pkn1 deficiency (Ldlr-KO;EC-Pkn1-KO). En face aortic arch preparations were stained with anti-CD31, anti-VCAM1 (**a**) or anti-SELE antibodies (**b**) as well as with DAPI. Immunofluorescence staining was quantified as the percentage of VCAM1-positive or SELE-positive endothelial cells (ECs) among CD31-positive cells per view field (*n* = 5 mice per condition; at least three areas were analyzed per animal). **c**–**i**, Atherosclerosis-prone *Ldlr*^−/−^ mice without (Ldlr-KO) or with endothelium-specific PKN1 deficiency (Ldlr-KO;EC-Pkn1-KO) underwent partial carotid artery ligation. Seven days after ligation, cross-sections of the left common carotid artery (ligated artery) were stained with antibodies against H3.3S31P (**c**), H3K27Ac (**d**) or H3K36me3 (**e**) and against CD31 as well as with DAPI. **f**, The staining for H3.3S31P, H3K27Ac or H3K36me3 was quantified by determining the percentage of positively stained cells per field of view (*n* = 5 mice per condition; at least three sections were analyzed per animal). **g**, Relative expression of mRNAs encoding FOS and FOSB in ECs of carotid arteries 7 days after ligation analyzed by qRT–PCR (*n* = 5 mice per group). **h**,**i**, Twenty-eight days after ligation, light microscopical images of carotid arteries (**h**) were taken, or en face immunofluorescence staining for the expression of VCAM1 in ECs was performed (**i**). Bar diagrams show the statistical evaluation (*n* = 8 (**h**) and *n* = 5 (**i**) mice per group). **j**–**l**, *Ldlr*^−/−^ mice without (Ldlr-KO) or with induced endothelium-specific Pkn1 deficiency (Ldlr-KO;EC-Pkn1-KO) were fed an HFD for 16 weeks. Thereafter, aortas and adjacent vessels were analyzed. Shown are representative images of atherosclerotic plaques observed in brachiocephalic arteries (innominate arteries) (**j**) of Oil Red O-stained atherosclerotic lesions in the aortic valve region (**k**) and of whole aortas prepared en face and stained with Oil Red O (**l**) (*n* = 9 mice per group). Bar lengths, 50 µm (**a**,**b**), 20 µm (**c**–**e**), 5 mm (**h**,**l**), 100 µm (**i**,**j**) and 250 µm (**k**). Data are shown as mean ± s.e.m.; the *P* values are given in the figure (unpaired two-sided *t*-test (**a**,**b**,**f**,**h**–**l**) and Kruskal–Wallis test (**g**)). NS, not significant. Source data

**Extended Data Fig. 7:**
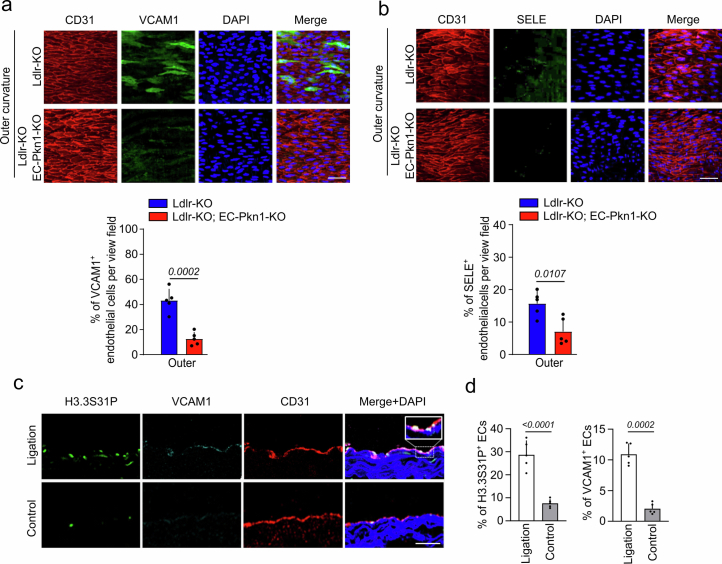
PKN1 mediates endothelial inflammation and flow-dependent histone H3.3 phosphorylation *in vivo.* **a**, **b**, Shown are representative en face immuno-confocal microscopy images of the outer curvature from 12-week-old atherosclerosis-prone *Ldlr*^-/-^ mice without (Ldlr-KO) or with endothelium-specific *Pkn1* deficiency (Ldlr-KO;EC-Pkn1-KO). En face aortic arch preparations were stained with antibodies against CD31, VCAM1 (a) or SELE (b) as well as with DAPI (a, b). Immunofluorescence staining was quantified as the percentage of VCAM1- or SELE-positive cells among CD31-positive cells per view field (*n* = 5 mice per group; at least 3 areas were analyzed per animal). **c**, **d**, 1 day after partial carotid ligation, immunofluorescence staining for the phosphorylation of histone H3.3 at S31 or for expression of VCAM1 in endothelial cells was performed using specific antibodies. Shown are representative sections of the ligated and unligated (control) carotid artery (c) and the statistical analysis (*n* = 5; d). Bar length: 50 µm. Data are shown as mean ± s.e.m.; the p-values are given in the figure (unpaired two-sided *t* test (a,b,d)). Source data

To study the role of PKN1 in disturbed-flow-induced inflammatory signaling under in vivo conditions, we analyzed EC-Pkn1-KO animals crossed with LDL receptor (Ldlr)-deficient mice. In the inner and outer curvature of the aortic arch, endothelial VCAM1 and SELE protein levels were reduced in mice with endothelium-specific PKN1 deficiency (Fig. 6a,b and Extended Data Fig. 7a,b). To analyze the functional consequences of endothelial PKN1 deficiency under pathological conditions, we performed partial carotid artery ligation. Seven days after partial carotid artery ligation, we detected in the endothelium of wild-type mice acetylation of H3K27 and tri-methylation of H3K36 as well as H3.3S31 phosphorylation, which was already observed 1 day after the ligation (Fig. 6c–f and Extended Data Fig. 7c,d). However, in endothelial cells of EC-Pkn1-KO animals, these levels were reduced (Fig. 6c–f). Consistent with this, expression of *Fos* and *Fosb* increased in endothelial cells of the partially ligated carotid arteries from wild-type mice but not in those from EC-Pkn1-KO animals (Fig. 6g). Twenty-eight days after partial carotid artery ligation, EC-Pkn1-KO mice showed reduced vascular remodeling and endothelial inflammation (Fig. 6h,i). Feeding of control mice and EC-Pkn1-KO animals lacking the Ldlr with a high-fat diet (HFD) for 16 weeks led to the development of atherosclerosis with strongly reduced plaque sizes in the aorta, in the brachiocephalic artery as well as in the outflow tract of EC-Pkn1-KO mice compared to control animals (Fig. 6j–l).

### Phosphorylation of H3.3S31 by PKN1 in macrophages

**Fig. 7:**
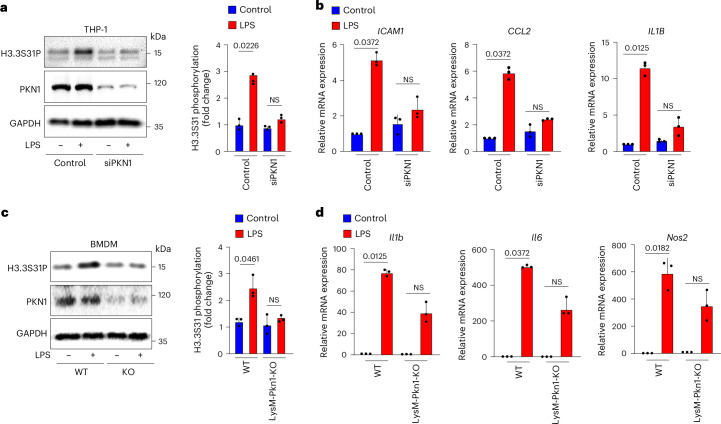
Phosphorylation of H3.3S31 by PKN1 in macrophages. **a**,**b**, THP-1 cells were transfected with a control siRNA or siRNAs directed against *PKN1*. Phosphorylated H3.3S31, PKN1 and GAPDH in lysates were determined by immunoblotting (**a**). Inflammatory gene expression in response to LPS (1 µg ml^−1^, 1 h) was analyzed by qRT–PCR (**b**) (*n* = 3 independent experiments). **c**,**d**, BMDMs were isolated from wild-type (WT) or LysM-Cre;*Pkn1*^flox/flox^ (KO) mice and were stimulated for 1 h with 1 µg ml^−1^ LPS. Phosphorylated H3.3S31, PKN1 and GAPDH in lysates were determined by immunoblotting (**c**). Inflammatory gene expression was analyzed by qRT–PCR (**d**) (*n* = 3 mice per group). Data are shown as mean ± s.e.m.; the *P* values are given in the figure (Kruskal–Wallis test (**a**–**d**)). NS, not significant. Source data

Because our data identified PKN1 in endothelial cells as the kinase mediating acute disturbed-flow-induced expression of inflammatory genes by PKN1-mediated H3.3S31 phosphorylation, which was shown to play an important role also in the acute activation of other cell types, including stem cells and macrophages^32,34^, we tested whether the PKN1-mediated phosphorylation of serine 31 is a more general mechanism, by which H3.3 serine 31 is phosphorylated and, thereby, controls cell activation. We, therefore, studied macrophages, which use H3.3S31 phosphorylation as a mechanism to mediate rapid transcription in response to activation by LPS^34^. In human monocytic THP-1 cells in which PKN1 was knocked down by transfection with an siRNA directed against *PKN1*, LPS lost the ability to induce H3.3 serine 31 phosphorylation compared to cells transfected with a control siRNA (Fig. 7a). In addition, LPS-induced expression of inflammatory genes, such as *ICAM1*, *CCL2* and *IL1B*, was reduced (Fig. 7b). To analyze the potential role of PKN1 on LPS-induced H3.3 serine 31 phosphorylation in bone-marrow-derived macrophages (BMDMs), we generated mice lacking PKN1 in myeloid cells by crossing mice carrying the floxed *Pkn1* allele with the LysM-Cre mouse line^46^. In isolated BMDMs, we found that LPS-induced H3.3S31 phosphorylation was compromised compared to BMDMs from control animals (Fig. 7c). The reduced H3.3S31 phosphorylation in response to LPS in BMDMs from myeloid-cell-specific PKN1-deficient mice was accompanied by reduced LPS-induced gene induction (Fig. 7d). These data show that PKN1 is also involved in mediating H3.3S31 phosphorylation and subsequent gene expression in macrophages.

## Discussion

In contrast to canonical histone H3, the histone variant H3.3 is expressed throughout the cell cycle and in quiescent cells^31,40^. H3.3 is characteristic of euchromatin and has preferentially been found at transcriptionally dynamic regions of the genome, such as enhancers, promoters and gene bodies^47–50^. Consistent with this, it is enriched in post-translational histone modifications characteristic for active chromatin states^51,52^. H3.3 differs only in a few amino acid residues from canonical H3, namely H3.1 and H3.2, including a serine residue in position 31, which is an alanine in canonical H3. Several recent studies indicated that this serine residue and its phosphorylation state influence chromatin states at active regulatory elements and genes. In ESCs, H3.3S31 phosphorylation promotes active chromatin states, such as H3K27ac, by stimulating CBP/p300 histone acetyl transferase activity^32^. H3.3S31 was shown to be rapidly phosphorylated in various immune cells activated through different receptors as well as in neurons in response to BDNF^34^. In activated macrophages, H3.3S31 phosphorylation leads to the ejection of the transcriptional repressor ZMYND11 and enables recruitment of the H3K36 methyltransferase SETD2, which, in turn, increases its activity and leads to rapid and robust transcription^34^. Our data show that atherogenic disturbed flow induces H3.3S31 phosphorylation in endothelial cells to induce H3K27ac and H3K36me3 chromatin modifications in gene bodies and promoter regions of the AP-1 transcription factors FOS and FOSB to promote their rapid expression, which is a requirement for flow-induced endothelial expression of inflammatory genes. We, thereby, identify a critical mechanism of inflammatory gene induction in the endothelium and also demonstrate that H3.3S31 phosphorylation is a widely used mechanism to mediate rapid signaling-induced gene activation (Fig. 5j).

Several kinases are able to mediate H3.3S31 phosphorylation. During mitosis, checkpoint kinase 1 (CHK1, CHEK1) and aurora B kinase can phosphorylate H3.3S31 (refs. ^41,53^). CHK1 was also shown to be responsible for H3.3S31 phosphorylation in the euchromatin of mouse ESCs, resulting in H3K27 acetylation at enhancers by CBP/p300, which facilitates differentiation of ESCs^32^. Also, IκB kinase α (IKKα) can phosphorylate H3.3S31 in transcribing regions^42^, and IKKα was shown to be involved in acute stimulus-induced phosphorylation of H3.3S31 and subsequent SETD2-mediated H3K36 tri-methylation to enable rapid gene transcription^34^. We provide evidence that also PKN1 can directly phosphorylate H3.3S31 and that PKN1, but not CHK1 or IKKα, mediates disturbed-flow-induced H3.3S31 phosphorylation to promote acute induction of *FOS*/*FOSB* expression and subsequent expression of inflammatory genes. Thus, H3.3S31 phosphorylation appears to be a critical step during acute gene transcription in various cellular systems and physiological contexts, and, depending on the cell type and upstream stimulus, different kinases can mediate H3.3S31 phosphorylation.

NF-κB and AP-1 are major transcription factors activated by disturbed flow^11,16^. Our data show that disturbed-flow-induced NF-κB activation is independent of PKN1, whereas PKN1 plays a critical role in AP-1 activation in response to disturbed flow by mediating JUN phosphorylation and disturbed-flow-induced induction of *FOS/FOSB*. It was previously shown that fluid shear stress induces JNK activation^15,54,55^ and that this is mediated by integrins, including integrin α5β1, through the activation of a cascade of kinases, including mitogen-activated protein kinase kinase 4 (MKK4) and p21-activated kinase (PAK)^54^. We do not know how integrin α5β1 activates PKN1, but it appears likely that integrin-dependent activation of PKN1 is part of this integrin-induced protein kinase cascade, which may also explain the regulation of JNK by PKN1.

Although NF-κB and AP-1 are regulated independently by disturbed flow, both are required for disturbed-flow-induced expression of inflammatory genes^16^. This indicates that NF-κB and AP-1 cooperate, and cooperative activation of both transcription factors was shown in numerous cells, including endothelial cells^56^. The cooperation between NF-κB and AP-1 is based on their direct interaction^57^, which results in the formation of a physical complex between both transcription factors, which, in turn, enhances the functional activity of both NF-κB and AP-1 (ref. ^57^). Disturbed-flow-induced inflammatory gene expression, which requires both NF-κB and AP-1 activation, is, therefore, likely to depend on the cooperation of both transcription factors by direct physical interaction or by other indirect mechanisms^58,59^.

PKN1 was previously shown to translocate to the nucleus in response to cell stress^60^ and to induce histone phosphorylation at H3T11 during androgen stimulation^39^. H3T11 phosphorylation by PKN1 leads to enhancement of the demethylation of H3K9me3 by the histone demethylase JMJD2C at androgen-responsive genes and promotes transcription initiation^39^. In addition, evidence was provided that PKN1-mediated H3T11 phosphorylation leads to the recruitment of the WD repeat-containing protein 5 (WDR5) component of the SET/mixed-lineage leukemia (MLL) histone methyl transferase complex to androgen-responsive elements, resulting in tri-methylation of H3K4 (ref. ^61^). How PKN1 is recruited and activated to induce H3T11 phosphorylation has, however, remained unclear. Our study extends the spectrum of histone sites phosphorylated by PKN1 and shows that, in endothelial cells, PKN1 is a central kinase mediating disturbed-flow-induced H3.3S31 phosphorylation. We also show that PKN1 translocates to the nucleus in an integrin α5β1-dependent manner. Rapid induction of gene transcription through PKN1-mediated phosphorylation of H3.3S31 appears to be a more general phenomenon, as we could show that LPS-induced H3.3S31 phosphorylation and subsequent gene transcription in human THP1 macrophages, as well as in murine BMDMs, also involve PKN1.

## Methods

### Reagents

Antibodies directed against phosphorylated PKN1 (T774; cat. no. 2611, 1:1,000), FOS (cat. no. 2250, 1:1,000), FOSB (cat. no. 2251, 1:1,000), GAPDH (cat. no. 2118, 1:3,000), phosphorylated c-JUN (S63; cat. no. 91952, 1:1,000), JUN (cat. no. 9165, 1:1,000), phosphorylated JNK (T183/T185; cat. no. 9251, 1:1,000), tubulin (cat. no. 2125, 1:2,000), lamin A/C (cat. no. 2032, 1:1,000) phosphorylated P65 (S536; cat. no. 3033, 1:1,000) and P65 (cat. no. 4764, 1:1,000) were obtained from Cell Signaling Technology. Antibodies against ICAM1 (cat. no. ab119871, 1:500), mouse VCAM1 (cat. no. ab134047, 1:500), mouse CD31 (cat. no. ab24590, 1:500), histone H3S10P (cat. no. ab5176, 1:1,000), histone H3T11P (cat. no. ab5168, 1:1,000), histone H3S28P (cat. no. ab32388, 1:1,000), histone H3.3S31P (cat. no. ab92628, 1:1,000), histone H3.3. (cat. no. ab176840, 1:1,000) and histone H3K36me3 (cat. no. ab9050, 1:1,000) were obtained from Abcam. The anti-histone H3K27 (cat. no. 39133, 1:1,000) and anti-histone H3.1/2 (cat. no. 61629, 1:1,000) antibodies were obtained from Active Motif. The human anti-VCAM1 (cat. no. BBA19, 1:300) and human anti-CD31 (cat. no. AF806, 1:300) antibodies were obtained from Bio-Techne. The anti-PKN1 antibody (cat. no. 610687, 1:1,000) was obtained from BD Biosciences, and an antibody against phosphorylated PKN1 (cat. no. AB-PK781, 1:1,000) was obtained from Kinexus Bioinformatics Corporation. ATN161 (cat. no. 6058) was obtained from R&D Systems. Fibronectin (cat. no. F1141), poly-l-lysine (cat. no. P8920), collagen IV (cat. no. CC076) and LPS (cat. no. L2630) were obtained from Sigma-Aldrich. TNF (cat. no. AF- 300-01A-50) was obtained from PeproTech.

### Cells and cell culture

HUAECs were purchased from Provitro AG. Cells were cultured in EGM-2 MV medium (Lonza). HEK293T cells were purchased from the American Type Culture Collection and cultured in DMEM high (Invitrogen) containing 10% FBS. THP-1 cells were purchased from Sigma-Aldrich (cat. no. 88081201) and cultured in RPMI 1641 (Invitrogen) containing 10% FBS. To obtain BMDMs, the bone marrow of mouse femora and tibiae was flushed out with DMEM high-glucose medium using a 27-gauge needle. BMDMs were then prepared and cultured as previously described^62^.

### siRNA-mediated knockdown

Endothelial cells were transfected with siRNA using Opti-MEM (Thermo Fisher Scientific) and Lipofectamine RNAiMAX (Invitrogen) as described previously^63^. FOS esiRNA (EHU034291), FOSB esiRNA (EHU034891), P300 esiRNA (EHU155151), SETD2 esiRNA (EHU075161), H3F3B esiRNA (EHU109921) and control siRNA (no. SIC001) were purchased from Sigma-Aldrich. siRNAs used for the protein kinase screen were pools of three siRNAs directed against protein kinases that show high expression in endothelial cells and were obtained from Sigma-Aldrich. The targeted sequences of the siRNAs are shown in Supplementary Table 1.

### Shear stress assays

Endothelial cells were seeded on µ-Slide I Luer (ibidi, cat. no. 80176), and oscillatory flow (±4 dynes/cm^2^, 1 Hz) was applied to confluent monolayers using the ibidi pump system chamber as described previously^21^. The BioTech-Flow System cone-plate viscosimeter (MOS Technologies) was used for experiments to be analyzed by RNA sequencing (RNA-seq) and ATAC-seq as well as by ChIP assay. Cells were exposed to disturbed flow at ±4 dynes/cm^2^ (at a rotation speed of 28 r.p.m., 40 amplitude and 1 Hz). Shear stress was calculated with the following formula (assuming a Reynolds number of <1): *τ* = *η* × 2π × *n*/0.044 (*τ*, shear stress; *η*, viscosity; *n*, rotational speed^64^).

### Western blotting and subcellular fractionation

Endothelial cells were lysed in RIPA buffer (0.05 M Tris-HCl, pH 7.4, 0.15 M NaCl, 0.25% deoxycholic acid, 1% NP-40, 1 mM EDTA) (Sigma-Aldrich, cat. no. 89900) freshly supplemented with protease and phosphatase inhibitors (Roche, PhosSTOP). Total cell lysates were subjected to SDS-PAGE electrophoresis and were transferred to PVDF membranes. After blocking with 5% BSA for 1 h, the membranes were incubated with primary antibodies overnight at 4 °C. Thereafter, membranes were incubated with horseradish peroxidase (HRP)-conjugated secondary antibodies (Cell Signaling Technology, dilution 1:3,000) for 1 h at room temperature, followed by chemiluminescence detection using ECL Substrate (Pierce) according to the manufacturer’s protocol. Cytoplasmic and nuclear protein fractions were prepared using the NE-PER Nuclear and Cytoplasmic Extraction Kit (Pierce). The intensities of protein bands were quantified using the ImageJ Gel Analysis program (2.16.0) (National Institutes of Health).

### In vitro phosphorylation assay

Kinase activity was determined by performing an in vitro kinase assay^65^. For in vitro kinase assay using PKN1, recombinant human histone H3.3 (Sigma-Aldrich, cat. no. H2542-100UG), recombinant human H4 (Sigma-Aldrich, cat. no. H2667-100UG) or recombinant human histone H3.3 polynucleosomes (Active Motif, cat. no. 31468) and recombinant PKN1 (BIOZOL, cat. no. SCM-P70-11G-10) were incubated for 30 min at 30 °C in 20 μl of kinase buffer (25 mM MOPS, pH 7.2, 12.5 mM β-glycerol-phosphate, 25 mM MgCl_2_, 5 mM EGTA, 2 mM EDTA, 0.25 mM DTT, 50 µM ATP), which, in the indicated cases, contained 20 μCi (γ-^32^P)-ATP (Hartmann Analytic). To stop the reaction, SDS-PAGE loading buffer was added. The reaction mixture was separated by SDS-PAGE, and histone H3.3 phosphorylation at serine 31 was detected by immunoblotting using the anti-phospho-histone H3.3S31 antibody, and ^32^P-labeled proteins were visualized by autoradiography.

### Animal models

All mice were backcrossed onto a C57BL/6N background at least 8–10 times, and experiments were performed with littermates as controls. Male and female animals (8–12 weeks old) were used unless stated otherwise. Mice were housed under a 12-h light/dark cycle, with free access to food and water and under specific pathogen-free conditions unless stated otherwise. Mice carrying a floxed allele of the *Pkn1* gene were described previously^63,66^. The floxed *Itga5* allele was obtained from The Jackson Laboratory (stock no. 03299), and the endothelium-specific Cre transgenic lines Cdh5-CreERT2 and Tek-CreERT2 as well as the myeloid-cell-specific Cre line LysM-Cre were described previously^67,68^. Mice carrying floxed alleles but no Cre transgene were used as controls.

### Lentiviral infection of cells

The cDNA encoding human wild-type or mutant forms of histone H3.3 and constitutively active PKN1 (PKN1-CA, amino acids 561–942)^69^ were cloned into the lentiviral pLVX-IRES-ZsGreen1 expression vector (Clontech) and were used to transfect HEK293T cells along with the envelope plasmid pMD2.G and packaging plasmid psPAX2 using Lipofectamine 3000 Transfection Reagent (Thermo Fisher Scientific) according to the manufacturer’s protocol. After 48 h, supernatants containing lentiviral particles were harvested and filtered through a 0.45-µm low protein binding Durapore membrane (Millex) to remove cell debris. Before transduction, HUAECs were transfected with siRNA against human H3.3 using Lipofectamine RNAiMAX (Invitrogen). For lentiviral transduction, HUAECs were seeded in six-well plates, and the concentrated lentivirus was added at a multiplicity of infection (MOI) of 10. After 48 h, cells were used for further analyses.

### AAV infection

AAV2-QuadYF^70^ carrying the cDNA encoding mouse wild-type histone H3.3 or the H3.3(S31A) mutant was generated by VectorBuilder (Cyagen Biosciences). To generate AAV2-QuadYF viral particles, constructs encoding wild-type histone H3.3 or the corresponding H3.3S31A mutant fused C-terminally with EGFP and under the control of the murine *Tie1* promoter were used (Extended Data Fig. 5d). Then, 10–12-week-old *Ldlr*^−/−^ mice were briefly anesthetized by isoflurane inhalation, and AAV2-QuadYF H3.3 wild-type or H3.3 (S31A) (1 × 10^11^ viral genomes in 100 μl of saline) was injected intravenously.

### qRT–PCR analysis

Total RNA was isolated using an RNeasy Micro Kit (Qiagen) according to the manufacturer’s instructions. Reverse transcription was performed using the ProtoScript II Reverse Transcription Kit (New England Biolabs, M0368S). In brief, to synthesize cDNA, 1 µg of total RNA was combined with 0.5 µg of random hexamer primers (Roche), 0.5 mM dNTPs, 10 mM DTT, ProtoScript II Reverse Transcriptase (New England Biolabs) and RNase inhibitor (New England Biolabs). The resulting cDNA was used as a template for qPCR reactions using the LightCycler 480 Probe Master System (Roche) following the manufacturer’s protocol. The primer sequences are listed in Supplementary Table 2. The resulting Cq values were normalized to the reference gene *GAPDH*. The sequences of primers are shown in Supplementary Table 2.

### ChIP assay

HUAECs were seeded in a flow chamber of the BioTech-Flow System (MOS Technologies) and were exposed to disturbed flow as described above. Cells were fixed at 37 °C with 1% paraformaldehyde (PFA) for 15 min, followed by 0.2 M glycine for 5 min at room temperature. Thereafter, cells were washed twice with ice-cold PBS including protease inhibitors (Cell Signalling Technology, cat. no. 7012). Then, 5% of the cells were taken for DNA input analysis. With the rest, ChIP assay was performed using a SimpleChIP Enzymatic Chromatin IP Kit (Cell Signaling Technology, cat. no. 9003S) following the manufacturer’s instructions using antibodies directed against H3K27Ac or H3K36me3. The following primer pairs were used: *FOS* promoter primer (forward 5′- GAGCCCGTGACGTTTACACT-3′; reverse 5′-GCGAGCATCTGAGAAGCCAAGA-3′) and *FOSB* promoter primer (forward 5′-GCCGGAGATTTGGGGAAGTT-3′; reverse 5′-TAAGACTCAGAGCTACGGCCA-3′). *FOS* gene body primer (+444/+521: forward 5′-GTGCCTGGAGGGAGGCTGCCGT-3′; reverse 5′-GGGAACCAATTCTTACTATGGCA-3′; +1,443/+1,521: forward 5′-ACTGATGGGGCTGGCTGCACATC-3′; reverse 5′-ATGAGAGTACTTCTTAGGGTG-3′; +2,078/+2,157: forward 5′-ACTAGAGTTCATCCTGGCAGC-3′; reverse 5′-CCCAGTCAGATCAAGGGAAGC-3′; +2,669/+2,769: forward 5′-CTGCCCACCGCAAGGGCAGCA-3′; reverse 5′-CAGTGGCACTTGTGGGTGCCG-3′). *FOSB* gene body primer (+1/+140: forward 5′-ATTCATAAGACTCAGAGCT-3′; reverse 5′-ATCTTTCCAAAAACTTTCT-3′; +500/+600: forward 5′-CCCGGACTTGCACCTTACTT-3′; reverse 5′-CGTAGTCTCCGGGGAAAGCCT-3′; +1,000/+1,100: forward 5′-GGGGCTGAGAACTTTGAGCCG-3′; reverse 5′-AAAGGAAAAGAGACTGCTGGA-3′; +1,500/1,600: forward 5′-GCGGCTGGGTCTCTTTTCGGC-3′; reverse 5′-GAGCCCAGGGACCAGATCCCC-3′; +2,700/+2,800: forward 5′-AGGACCTCCAGTGGCTT-3’; reverse 5′-TCGTAGGGGTCGACGAC-3′).

### AP-1 luciferase reporter assay

HUAECs were seeded in a flow chamber of the BioTech-Flow System and grown to 60% confluence. Cells were then transfected with an AP-1 promoter luciferase reporter construct (3×AP-1 in pGL3-basic; Addgene, no. 40342). In brief, 500 ng of DNA and 3 μl of FuGENE reagent were diluted in 100 μl of Opti-MEM (Thermo Fisher Scientific). After incubation for 15 min, the mixture was added to HUAECs and then incubated for another 4 h. Thereafter, the medium was replaced by EGM-2 MV medium, and cells were incubated for 48 h. Cells were then exposed to either static or disturbed flow as described above. Luciferase activity in cell lysates was determined using the Dual-Luciferase Assay System (Promega, cat. no. E1910) according to the manufacturerʼs instructions.

### Immunohistochemistry of human aorta

Human aortic samples were obtained from patients undergoing dissecting aorta replacement surgery at the First Affiliated Hospital of Xi’an Jiaotong University. The study was approved by the ethics committee of Xi’an Jiaotong University (XJTU2018-249 and XJTU2019-12) and conforms to the guidelines of the 2000 Declaration of Helsinki. Written informed consent was obtained from all individuals before their participation. Between 3-mm-long and 25-mm-long segments of the aortic arch or descending aorta tissue were identified by using a silk thread, collected during surgery and immediately processed for fixation in 4% PFA. The samples were then permeabilized and incubated with antibodies directed against CD31 (R&D Systems, cat. no. AF806), PKN1 (Santa Cruz Biotechnology, cat. no. sc-271594), VCAM1 (Abcam, cat. no. ab134047) and phospho-H3.3S31 (Thermo Fisher Scientific, cat. no. 44-244G) and co-stained with DAPI (Invitrogen, cat. no. D1306). As secondary antibodies, Alexa Fluor 594 anti-sheep, Alexa Fluor 488 anti-mouse and Alexa Fluor 647 anti-rabbit (Thermo Fisher Scientific, cat. nos. A-11016, A-11001, A-21443 and A-21244, respectively) were used. Tissue was mounted in mounting medium (Polysciences, cat. no. 18606-5), and z-stack projection was acquired using a confocal microscope (Leica, SP8 MP). Image analysis was performed with ImageJ.

### Partial carotid artery ligation

Partial carotid artery ligation was performed as described previously^71^ with modifications^72^. In brief, male mice were anesthetized by intraperitoneal injection of 120 mg kg^−1^ ketamine (Pfizer) and 16 mg kg^−1^ xylazine (Bayer) and were placed on a heated surgical pad. After hair removal, a midline cervical incision was made, and the left internal and external carotid arteries were exposed and partially ligated with 6-0 silk sutures (Serag-Wiessner), leaving the superior thyroid artery intact. Skin was sutured with absorbable 6-0 silk suture (CatGut), and animals were monitored until recovery in a chamber on a heating pad after the surgery. Animals were fed an HFD for 1 week (Ssniff, cat. no. TD88137), at which time they were euthanized and arteries were isolated. The extent of carotid artery remodeling was quantified using ImageJ software and displayed as the percentage of carotid artery containing visible neointima between its origin at the aorta and the ligation site. The right, non-ligated carotid artery served as a control. For analysis of gene expression in endothelial cells, the carotid lumen was flushed with 150 μl of QIAzol lysis reagent (Qiagen) using a 29-gauge syringe, and the eluate was collected in a microtube. RNA isolation was performed using an miRNeasy Mini Kit (Qiagen) according to the manufacturer’s instructions, and mRNA was quantified by qRT–PCR.

### Histology and immunostaining

Mice were perfused with saline containing heparin (40 U ml^−1^), followed by 4% PFA in PBS. The mouse aorta was isolated and fixed with 4% PFA for 30 min at room temperature. Mouse aortas were incubated with 0.2% Triton X-100 and 1% BSA in PBS for 1 h at room temperature. The outer and inner curvature of aortas were dissected. Samples were incubated at 4 °C overnight in PBS containing primary antibodies (dilution 1:100) directed against CD31 (Abcam, cat. no. ab24590), VCAM1 (BD Pharmingen, clone 429, 550547) and SELE (BioVision, cat. no. 3631-100). After washing three times in PBS, aortas were incubated with Alexa Fluor 488-conjugated and Alexa Fluor 594-conjugated secondary antibodies (1:200; Invitrogen) together with DAPI (1 ng ml^−1^; Invitrogen) for 1 h at room temperature. After washing three times with PBS, tissues were mounted en face with Fluoromount (Sigma-Aldrich, cat. no. F4680) for confocal imaging.

For en face Oil Red O staining of atherosclerotic plaques, aortas were fixed in 4% PFA overnight at 4 °C after perfusion (4% PFA, 20 mM EDTA, 5% sucrose in 15 ml of PBS). Thereafter, connective tissue and the adventitia were removed, and vessels were cut, opened and pinned en face onto a glass plate coated with silicon. After rinsing with distilled water for 10 min and subsequently with 60% isopropanol, vessels were stained en face with Oil Red O for 30 min under gentle shaking and were then rinsed again in 60% isopropanol and then in tap water for 10 min. Samples were mounted on coverslips with the endothelial surface facing upwards with glycerol gelatin aqueous mounting media (Sigma-Aldrich). Images were acquired using a Leica LAS AF Lite microscope. For morphological analysis of atherosclerotic plaques, the brachiocephalic artery and the aortic sinus area attached to the heart were dissected and embedded in optimal cutting temperature (OCT) compound (Tissue-Tek). Frozen brachiocephalic arteries were mounted in a cryotome, and a defined segment 500–1,000 μm distal from the origin of the brachiocephalic artery was sectioned (10 μm). Sections were then stained with Oil Red O for 30 min and mounted with glycerol gelatin (Sigma-Aldrich, cat. no. 1092420100). To analyze the endothelial layer of the inner and outer aortic curvature, aortic arches were dissected and embedded in OCT compound. Frozen samples were cryosectioned (10 μm) and fixed with ice-cold acetone for 10 min. OCT compound was removed by washing with PBS three times for 5 min at room temperature, and sections were immunostained with antibodies against CD31 (BD Biosciences, cat. no. 550274) or phosphorylated PKN1 (Sigma-Aldrich, cat. no. F3648) overnight at 4 °C. After washing three times with PBS, bound primary antibodies were detected using Alexa Fluor 488-conjugated or Alexa Fluor 594-conjugated secondary antibodies (Invitrogen, cat. nos. A21206 and A21209; 1:200). DAPI (Invitrogen, cat. no. D3571; 1 ng ml^−1^) was used to label cell nuclei. Sections were viewed with a confocal microscope (Leica SP5 FLIM and Leica SP8 MP). For quantification, the endothelial cell area was defined by CD31 staining using ImageJ software, and the fluorescence signal indicating phospho-PKN1 was then calculated as percentage of total endothelial cell area.

### RNA-seq

RNA was extracted from HUAECs using an miRNeasy Micro Kit (Qiagen) combined with on-column DNase digestion (Qiagen, DNase-Free DNase Set). RNA and library preparation integrity were verified using the LabChip GX Touch 24 (PerkinElmer). Then, 3 µg of total RNA was used as input for TruSeq Stranded mRNA library preparation following the low sample protocol (Illumina). Sequencing was performed with the NextSeq 500 sequencing system (Illumina).

Trimmomatic was employed to trim reads after a quality drop below a mean of Q15 in a window of 5 nucleotides (nt) and keeping only filtered reads longer than 15 nt (ref. ^73^). Reads were aligned versus Ensembl human genome version hg38 (Ensembl release 104) with STAR^74^. Aligned reads were filtered to remove multi-mapping, ribosomal or mitochondrial reads. Gene counts were established with featureCounts by aggregating reads overlapping exons on the correct strand, excluding those overlapping multiple genes^75^. The raw count matrix was normalized with DESeq2 (ref. ^76^). Contrasts were created with DESeq2 based on the raw count matrix. Genes were classified as significantly differentially expressed at average count >5, multiple testing adjusted *P* < 0.05 and −0.585 < log_2_fold change (FC) > 0.585. The Ensembl annotation was enriched with UniProt data (activities at the Universal Protein Resource (UniProt)).

### ATAC-seq

For ATAC-seq, HUAECs were freshly processed. In brief, 50,000 cells were centrifuged at 500*g* for 5 min at 4 °C and washed with PBS. The cell pellet was resuspended in 50 µl of lysis/transposition reaction (12.5 µl of THS-TD-Buffer, 2.5 µl of Tn5, 5 µl of 0.1% digitonin, 30 µl of water) and incubated at 37 °C for 30 min with occasional snap mixing. For purification of the DNA fragments, a MinElute PCR Purification Kit (Qiagen) was used. Amplification of library together with indexing primers was performed as described previously^77^. Libraries were mixed in equimolar ratios and sequenced on NextSeq 500 and NextSeq 2000 platforms (Illumina).

Trimmomatic was employed to trim reads after a quality drop below a mean of Q15 in a window of 5 nt and keeping only filtered reads longer than 15 nt (ref. ^73^). Reads were aligned versus Ensembl human genome version hg38 (Ensembl release 104) with STAR 2.7.10a^74^. Aligned reads were filtered to remove duplicates with Picard (Picard: a set of tools (in Java) for working with next-generation sequencing data in the BAM format) and spliced, multi-mapping, ribosomal or mitochondrial reads. Peak calling was performed with MACS false discovery rate (FDR) < 0.0001 (ref. ^78^). Peaks overlapping ENCODE blacklisted regions (known misassemblies and satellite repeats) were excluded. Remaining peaks were unified to represent a common set of regions for all samples, and counts were produced with bigWigAverageOverBed (UCSC Toolkit). The raw count matrix was normalized with DESeq2 (ref. ^76^). Peaks were annotated with the promoter (transcription start site (TSS) ± 5,000 nt) of the nearest gene based on Ensembl release 104. Contrasts were created with DESeq2 based on the normalized union peak matrix with all size factors set to 1. Peaks were classified as significantly differential at average count >10, −0.585 < log_2_FC > 0.585 and adjusted *P* < 0.05.

### TOBIAS

TOBIAS was used to perform ATAC-seq footprinting of known transcription factors^35^. In brief, TOBIAS corrects for Tn5 bias, calculates footprint scores from the distribution of Tn5 insertions and compares these with all predicted binding sites based on position weight matrices. All TOBIAS analyses were run with default parameters and the HOCOMOCO version 11 database as reference (https://doi.org/10.1093%2Fnar%2Fgkx1106).

### Statistics

All statistical analyses were performed using GraphPad Prism version 8.3.0 (GraphPad Software). All experimental values are presented as mean ± s.e.m. Data were tested for normality using the Shapiro–Wilk test. Statistical analyses between two groups were conducted using either an unpaired two-tailed Student’s *t*-test (parametric) or the Mann–Whitney *U*-test (non-parametric). Multiple group comparisons were conducted using parametric analysis with one-way ANOVA, followed by Tukey’s post hoc test. Additionally, comparisons among multiple experimental groups at different timepoints were performed using two-way ANOVA, followed by Bonferroni’s post hoc test. For non-parametric multiple group comparisons, the Kruskal–Wallis test was performed, followed by Dunn’s multiple comparison. A *P* value of less than 0.05 was considered to indicate statistical significance.

### Study approval

Studies using human samples were approved by the ethics committee of Xi’an Jiaotong University (XJTU2018-249 and XJTU2019-12) and conform to the guidelines of the 2000 Declaration of Helsinki. Written informed consent was obtained from all individuals before their participation. All procedures involving animal care and use in this study were approved by the local animal ethics committees (Regierungspräsidium Darmstadt (Germany) and the ethics committee of Xi’an Jiaotong University (China)).

### Reporting summary

Further information on research design is available in the Nature Portfolio Reporting Summary linked to this article.

## Supplementary information

Reporting Summary

Supplementary Tables 1 and 2 Sequences of siRNAs (Supplementary Table 1); sequences of primers for PCR (Supplementary Table 2).

## Source data

Source Data Figs. 1–7 and Extended Data Figs. 1–7 Statistical source data.

Source Data Figs. 1–7 and Extended Data Figs. 1–7 Unprocessed western blots.

## Data Availability

ATAC-seq and bulk RNA-seq data generated for this study have been deposited at the Gene Expression Omnibus under accession number GSE261756. Source data are provided with this paper.

## Code availability

Custom code has been deposited in a GitHub repository and is available at 10.5281/zenodo.14181510.

## References

[1] Libby, P. et al. Atherosclerosis. *Nat. Rev. Dis. Primers* **5**, 56 (2019). 31420554 10.1038/s41572-019-0106-z

[2] Bjorkegren, J. L. M. & Lusis, A. J. Atherosclerosis: recent developments. *Cell* **185**, 1630–1645 (2022). 35504280 10.1016/j.cell.2022.04.004 PMC9119695

[3] Herrington, W., Lacey, B., Sherliker, P., Armitage, J. & Lewington, S. Epidemiology of atherosclerosis and the potential to reduce the global burden of atherothrombotic disease. *Circ. Res.* **118**, 535–546 (2016). 26892956 10.1161/CIRCRESAHA.115.307611

[4] Yurdagul, A. Jr, Finney, A. C., Woolard, M. D. & Orr, A. W. The arterial microenvironment: the where and why of atherosclerosis. *Biochem. J.* **473**, 1281–1295 (2016). 27208212 10.1042/BJ20150844 PMC5410666

[5] Tamargo, I. A., Baek, K. I., Kim, Y., Park, C. & Jo, H. Flow-induced reprogramming of endothelial cells in atherosclerosis. *Nat. Rev. Cardiol.* **20**, 738–753 (2023). 37225873 10.1038/s41569-023-00883-1 PMC10206587

[6] Zarins, C. K. et al. Carotid bifurcation atherosclerosis. Quantitative correlation of plaque localization with flow velocity profiles and wall shear stress. *Circ. Res.* **53**, 502–514 (1983). 6627609 10.1161/01.res.53.4.502

[7] Davis, M. J., Earley, S., Li, Y. S. & Chien, S. Vascular mechanotransduction. *Physiol. Rev.* **103**, 1247–1421 (2023). 36603156 10.1152/physrev.00053.2021 PMC9942936

[8] Davies, P. F. Flow-mediated endothelial mechanotransduction. *Physiol. Rev.* **75**, 519–560 (1995). 7624393 10.1152/physrev.1995.75.3.519 PMC3053532

[9] Hahn, C. & Schwartz, M. A. Mechanotransduction in vascular physiology and atherogenesis. *Nat. Rev. Mol. Cell Biol.* **10**, 53–62 (2009). 19197332 10.1038/nrm2596 PMC2719300

[10] Aitken, C., Mehta, V., Schwartz, M. A. & Tzima, E. Mechanisms of endothelial flow sensing. *Nat. Cardiovasc. Res.* **2**, 517–529 (2023). 39195881 10.1038/s44161-023-00276-0

[11] Mohan, S., Mohan, N. & Sprague, E. A. Differential activation of NF-kappa B in human aortic endothelial cells conditioned to specific flow environments. *Am. J. Physiol.* **273**, C572–C578 (1997). 9277354 10.1152/ajpcell.1997.273.2.C572

[12] Nagel, T., Resnick, N., Dewey, C. F. Jr & Gimbrone, M. A. Jr. Vascular endothelial cells respond to spatial gradients in fluid shear stress by enhanced activation of transcription factors. *Arterioscler. Thromb. Vasc. Biol.* **19**, 1825–1834 (1999). 10446060 10.1161/01.atv.19.8.1825

[13] Feaver, R. E., Gelfand, B. D., Wang, C., Schwartz, M. A. & Blackman, B. R. Atheroprone hemodynamics regulate fibronectin deposition to create positive feedback that sustains endothelial inflammation. *Circ. Res.* **106**, 1703–1711 (2010). 20378855 10.1161/CIRCRESAHA.109.216283 PMC2891748

[14] Hajra, L. et al. The NF-κB signal transduction pathway in aortic endothelial cells is primed for activation in regions predisposed to atherosclerotic lesion formation. *Proc. Natl Acad. Sci. USA* **97**, 9052–9057 (2000). 10922059 10.1073/pnas.97.16.9052 PMC16820

[15] Cuhlmann, S. et al. Disturbed blood flow induces RelA expression via c-Jun N-terminal kinase 1: a novel mode of NF-κB regulation that promotes arterial inflammation. *Circ. Res.* **108**, 950–959 (2011). 21350211 10.1161/CIRCRESAHA.110.233841

[16] Nakajima, H. & Mochizuki, N. Flow pattern-dependent endothelial cell responses through transcriptional regulation. *Cell Cycle* **16**, 1893–1901 (2017). 28820314 10.1080/15384101.2017.1364324 PMC5638382

[17] Sun, X. et al. Activation of integrin α5 mediated by flow requires its translocation to membrane lipid rafts in vascular endothelial cells. *Proc. Natl Acad. Sci. USA* **113**, 769–774 (2016). 26733684 10.1073/pnas.1524523113 PMC4725528

[18] Yun, S. et al. Interaction between integrin α5 and PDE4D regulates endothelial inflammatory signalling. *Nat. Cell Biol.* **18**, 1043–1053 (2016). 27595237 10.1038/ncb3405 PMC5301150

[19] Budatha, M. et al. Inhibiting integrin α5 cytoplasmic domain signaling reduces atherosclerosis and promotes arteriogenesis. *J. Am. Heart Assoc.* **7**, e007501 (2018). 29382667 10.1161/JAHA.117.007501 PMC5850249

[20] Bhullar, I. S. et al. Fluid shear stress activation of IκB kinase is integrin-dependent. *J. Biol. Chem.* **273**, 30544–30549 (1998). 9804824 10.1074/jbc.273.46.30544

[21] Albarran-Juarez, J. et al. Piezo1 and G_q_/G_11_ promote endothelial inflammation depending on flow pattern and integrin activation. *J. Exp. Med.* **215**, 2655–2672 (2018). 30194266 10.1084/jem.20180483 PMC6170174

[22] Chen, J. et al. αvβ3 integrins mediate flow-induced NF-κB activation, proinflammatory gene expression, and early atherogenic inflammation. *Am. J. Pathol.* **185**, 2575–2589 (2015). 26212910 10.1016/j.ajpath.2015.05.013 PMC4597278

[23] Ajami, N. E. et al. Systems biology analysis of longitudinal functional response of endothelial cells to shear stress. *Proc. Natl Acad. Sci. USA* **114**, 10990–10995 (2017). 28973892 10.1073/pnas.1707517114 PMC5642700

[24] Andueza, A. et al. Endothelial reprogramming by disturbed flow revealed by single-cell RNA and chromatin accessibility study. *Cell Rep.* **33**, 108491 (2020). 33326796 10.1016/j.celrep.2020.108491 PMC7801938

[25] Chung, J. et al. Coxsackievirus and adenovirus receptor mediates the responses of endothelial cells to fluid shear stress. *Exp. Mol. Med.* **51**, 1–15 (2019). 31776326 10.1038/s12276-019-0347-7 PMC6881322

[26] Lee, J. Y. et al. Fluid shear stress regulates the expression of Lectin-like oxidized low density lipoprotein receptor-1 via KLF2-AP-1 pathway depending on its intensity and pattern in endothelial cells. *Atherosclerosis* **270**, 76–88 (2018). 29407891 10.1016/j.atherosclerosis.2018.01.038

[27] Maurya, M. R. et al. Longitudinal shear stress response in human endothelial cells to atheroprone and atheroprotective conditions. *Proc. Natl Acad. Sci. USA* **118**, e2023236118 (2021). 33468662 10.1073/pnas.2023236118 PMC7848718

[28] DebRoy, A. et al. Cooperative signaling via transcription factors NF-κB and AP1/c-Fos mediates endothelial cell STIM1 expression and hyperpermeability in response to endotoxin. *J. Biol. Chem.* **289**, 24188–24201 (2014). 25016017 10.1074/jbc.M114.570051 PMC4148850

[29] Martin, T., Cardarelli, P. M., Parry, G. C., Felts, K. A. & Cobb, R. R. Cytokine induction of monocyte chemoattractant protein-1 gene expression in human endothelial cells depends on the cooperative action of NF-χB and AP-1. *Eur. J. Immunol.* **27**, 1091–1097 (1997). 9174597 10.1002/eji.1830270508

[30] Keegan, P. M., Anbazhakan, S., Kang, B., Pace, B. S. & Platt, M. O. Biomechanical and biochemical regulation of cathepsin K expression in endothelial cells converge at AP-1 and NF-κB. *Biol. Chem.* **397**, 459–468 (2016). 26760306 10.1515/hsz-2015-0244 PMC9717675

[31] Szenker, E., Ray-Gallet, D. & Almouzni, G. The double face of the histone variant H3.3. *Cell Res.* **21**, 421–434 (2011). 21263457 10.1038/cr.2011.14 PMC3193428

[32] Martire, S. et al. Phosphorylation of histone H3.3 at serine 31 promotes p300 activity and enhancer acetylation. *Nat. Genet.* **51**, 941–946 (2019). 31152160 10.1038/s41588-019-0428-5 PMC6598431

[33] Sitbon, D., Boyarchuk, E., Dingli, F., Loew, D. & Almouzni, G. Histone variant H3.3 residue S31 is essential for *Xenopus* gastrulation regardless of the deposition pathway. *Nat. Commun.* **11**, 1256 (2020). 32152320 10.1038/s41467-020-15084-4 PMC7062693

[34] Armache, A. et al. Histone H3.3 phosphorylation amplifies stimulation-induced transcription. *Nature* **583**, 852–857 (2020). 32699416 10.1038/s41586-020-2533-0 PMC7517595

[35] Bentsen, M. et al. ATAC-seq footprinting unravels kinetics of transcription factor binding during zygotic genome activation. *Nat. Commun.* **11**, 4267 (2020). 32848148 10.1038/s41467-020-18035-1 PMC7449963

[36] Dong, Z. M., Brown, A. A. & Wagner, D. D. Prominent role of P-selectin in the development of advanced atherosclerosis in ApoE-deficient mice. *Circulation* **101**, 2290–2295 (2000). 10811597 10.1161/01.cir.101.19.2290

[37] Budatha, M., Zhang, J. & Schwartz, M. A. Fibronectin-mediated inflammatory signaling through integrin α5 in vascular remodeling. *J. Am. Heart Assoc.* **10**, e021160 (2021). 34472370 10.1161/JAHA.121.021160 PMC8649308

[38] Zhang, C. et al. Coupling of integrin α5 to annexin A2 by flow drives endothelial activation. *Circ. Res.* **127**, 1074–1090 (2020). 32673515 10.1161/CIRCRESAHA.120.316857

[39] Metzger, E. et al. Phosphorylation of histone H3 at threonine 11 establishes a novel chromatin mark for transcriptional regulation. *Nat. Cell Biol.* **10**, 53–60 (2008). 18066052 10.1038/ncb1668 PMC2878724

[40] Delaney, K., Weiss, N. & Almouzni, G. The cell-cycle choreography of H3 variants shapes the genome. *Mol. Cell* **83**, 3773–3786 (2023). 37734377 10.1016/j.molcel.2023.08.030 PMC10621666

[41] Chang, F. T. et al. CHK1-driven histone H3.3 serine 31 phosphorylation is important for chromatin maintenance and cell survival in human ALT cancer cells. *Nucleic Acids Res.* **43**, 2603–2614 (2015). 25690891 10.1093/nar/gkv104 PMC4357709

[42] Thorne, J. L., Ouboussad, L. & Lefevre, P. F. Heterochromatin protein 1 gamma and IκB kinase alpha interdependence during tumour necrosis factor gene transcription elongation in activated macrophages. *Nucleic Acids Res.* **40**, 7676–7689 (2012). 22649058 10.1093/nar/gks509 PMC3439902

[43] Malik, A. N. et al. Genome-wide identification and characterization of functional neuronal activity-dependent enhancers. *Nat. Neurosci.* **17**, 1330–1339 (2014). 25195102 10.1038/nn.3808 PMC4297619

[44] Chen, L. F. et al. Enhancer histone acetylation modulates transcriptional bursting dynamics of neuronal activity-inducible genes. *Cell Rep.* **26**, 1174–1188 (2019). 30699347 10.1016/j.celrep.2019.01.032 PMC6376993

[45] Edmunds, J. W., Mahadevan, L. C. & Clayton, A. L. Dynamic histone H3 methylation during gene induction: HYPB/Setd2 mediates all H3K36 trimethylation. *EMBO J.* **27**, 406–420 (2008). 18157086 10.1038/sj.emboj.7601967 PMC2168397

[46] Clausen, B. E., Burkhardt, C., Reith, W., Renkawitz, R. & Forster, I. Conditional gene targeting in macrophages and granulocytes using LysMcre mice. *Transgenic Res.* **8**, 265–277 (1999). 10621974 10.1023/a:1008942828960

[47] Mito, Y., Henikoff, J. G. & Henikoff, S. Genome-scale profiling of histone H3.3 replacement patterns. *Nat. Genet.* **37**, 1090–1097 (2005). 16155569 10.1038/ng1637

[48] Goldberg, A. D. et al. Distinct factors control histone variant H3.3 localization at specific genomic regions. *Cell* **140**, 678–691 (2010). 20211137 10.1016/j.cell.2010.01.003 PMC2885838

[49] Deaton, A. M. et al. Enhancer regions show high histone H3.3 turnover that changes during differentiation. *eLife* **5**, e15316 (2016). 27304074 10.7554/eLife.15316 PMC4965263

[50] Chen, P. et al. H3.3 actively marks enhancers and primes gene transcription via opening higher-ordered chromatin. *Genes Dev.* **27**, 2109–2124 (2013). 24065740 10.1101/gad.222174.113 PMC3850095

[51] Loyola, A., Bonaldi, T., Roche, D., Imhof, A. & Almouzni, G. PTMs on H3 variants before chromatin assembly potentiate their final epigenetic state. *Mol. Cell* **24**, 309–316 (2006). 17052464 10.1016/j.molcel.2006.08.019

[52] McKittrick, E., Gafken, P. R., Ahmad, K. & Henikoff, S. Histone H3.3 is enriched in covalent modifications associated with active chromatin. *Proc. Natl Acad. Sci. USA* **101**, 1525–1530 (2004). 14732680 10.1073/pnas.0308092100 PMC341768

[53] Li, M., Dong, Q. & Zhu, B. Aurora kinase B phosphorylates histone H3.3 at serine 31 during mitosis in mammalian cells. *J. Mol. Biol.* **429**, 2042–2045 (2017). 28137420 10.1016/j.jmb.2017.01.016

[54] Hahn, C., Orr, A. W., Sanders, J. M., Jhaveri, K. A. & Schwartz, M. A. The subendothelial extracellular matrix modulates JNK activation by flow. *Circ. Res.* **104**, 995–1003 (2009). 19286608 10.1161/CIRCRESAHA.108.186486 PMC2702158

[55] Takabe, W. et al. Oscillatory shear stress induces mitochondrial superoxide production: implication of NADPH oxidase and c-Jun NH_2_-terminal kinase signaling. *Antioxid. Redox Signal.* **15**, 1379–1388 (2011). 20919940 10.1089/ars.2010.3645 PMC3144427

[56] Rius-Perez, S., Perez, S., Marti-Andres, P., Monsalve, M. & Sastre, J. Nuclear factor kappa B signaling complexes in acute inflammation. *Antioxid. Redox Signal.* **33**, 145–165 (2020). 31856585 10.1089/ars.2019.7975

[57] Stein, B. et al. Cross-coupling of the NF-kappa B p65 and Fos/Jun transcription factors produces potentiated biological function. *EMBO J.* **12**, 3879–3891 (1993). 8404856 10.1002/j.1460-2075.1993.tb06066.x PMC413671

[58] Fujioka, S. et al. NF-κB and AP-1 connection: mechanism of NF-κB-dependent regulation of AP-1 activity. *Mol. Cell. Biol.* **24**, 7806–7819 (2004). 15314185 10.1128/MCB.24.17.7806-7819.2004 PMC507000

[59] Riedlinger, T. et al. NF-κB p65 dimerization and DNA-binding is important for inflammatory gene expression. *FASEB J.* **33**, 4188–4202 (2019). 30526044 10.1096/fj.201801638R PMC6404571

[60] Mukai, H. et al. Translocation of PKN from the cytosol to the nucleus induced by stresses. *Proc. Natl Acad. Sci. USA* **93**, 10195–10199 (1996). 8816775 10.1073/pnas.93.19.10195 PMC38360

[61] Kim, J. Y. et al. A role for WDR5 in integrating threonine 11 phosphorylation to lysine 4 methylation on histone H3 during androgen signaling and in prostate cancer. *Mol. Cell* **54**, 613–625 (2014). 24793694 10.1016/j.molcel.2014.03.043 PMC4075454

[62] Toda, G., Yamauchi, T., Kadowaki, T. & Ueki, K. Preparation and culture of bone marrow-derived macrophages from mice for functional analysis. *STAR Protoc.* **2**, 100246 (2021). 33458708 10.1016/j.xpro.2020.100246 PMC7797923

[63] Jin, Y. J. et al. Protein kinase N2 mediates flow-induced endothelial NOS activation and vascular tone regulation. *J. Clin. Invest.* **131**, e145734 (2021). 34499618 10.1172/JCI145734 PMC8553558

[64] Buschmann, M. H., Dieterich, P., Adams, N. A. & Schnittler, H. J. Analysis of flow in a cone-and-plate apparatus with respect to spatial and temporal effects on endothelial cells. *Biotechnol. Bioeng.* **89**, 493–502 (2005). 15648084 10.1002/bit.20165

[65] Powell, D. W., Rane, M. J., Chen, Q., Singh, S. & McLeish, K. R. Identification of 14-3-3ζ as a protein kinase B/Akt substrate. *J. Biol. Chem.* **277**, 21639–21642 (2002). 11956222 10.1074/jbc.M203167200

[66] Sakaguchi, T. et al. Protein kinase N promotes stress-induced cardiac dysfunction through phosphorylation of myocardin-related transcription factor A and disruption of its interaction with actin. *Circulation* **140**, 1737–1752 (2019). 31564129 10.1161/CIRCULATIONAHA.119.041019

[67] Korhonen, H. et al. Anaphylactic shock depends on endothelial G_q_/G_11_. *J. Exp. Med.* **206**, 411–420 (2009). 19171764 10.1084/jem.20082150 PMC2646572

[68] Sorensen, I., Adams, R. H. & Gossler, A. DLL1-mediated Notch activation regulates endothelial identity in mouse fetal arteries. *Blood* **113**, 5680–5688 (2009). 19144989 10.1182/blood-2008-08-174508

[69] Park, Y. H., Wood, G., Kastner, D. L. & Chae, J. J. Pyrin inflammasome activation and RhoA signaling in the autoinflammatory diseases FMF and HIDS. *Nat. Immunol.* **17**, 914–921 (2016). 27270401 10.1038/ni.3457 PMC4955684

[70] Lipinski, D. M. et al. Systemic vascular transduction by capsid mutant adeno-associated virus after intravenous injection. *Hum. Gene Ther.* **26**, 767–776 (2015). 26359319 10.1089/hum.2015.097 PMC4651034

[71] Nam, D. et al. Partial carotid ligation is a model of acutely induced disturbed flow, leading to rapid endothelial dysfunction and atherosclerosis. *Am. J. Physiol. Heart Circ. Physiol.* **297**, H1535–H1543 (2009). 19684185 10.1152/ajpheart.00510.2009 PMC2770764

[72] Liang, G. et al. Tenascin-X mediates flow-induced suppression of EndMT and atherosclerosis. *Circ. Res.* **130**, 1647–1659 (2022). 35443807 10.1161/CIRCRESAHA.121.320694

[73] Bolger, A. M., Lohse, M. & Usadel, B. Trimmomatic: a flexible trimmer for Illumina sequence data. *Bioinformatics* **30**, 2114–2120 (2014). 24695404 10.1093/bioinformatics/btu170 PMC4103590

[74] Dobin, A. et al. STAR: ultrafast universal RNA-seq aligner. *Bioinformatics* **29**, 15–21 (2013). 23104886 10.1093/bioinformatics/bts635 PMC3530905

[75] Liao, Y., Smyth, G. K. & Shi, W. featureCounts: an efficient general purpose program for assigning sequence reads to genomic features. *Bioinformatics* **30**, 923–930 (2014). 24227677 10.1093/bioinformatics/btt656

[76] Love, M. I., Huber, W. & Anders, S. Moderated estimation of fold change and dispersion for RNA-seq data with DESeq2. *Genome Biol.* **15**, 550 (2014). 25516281 10.1186/s13059-014-0550-8 PMC4302049

[77] Buenrostro, J. D., Giresi, P. G., Zaba, L. C., Chang, H. Y. & Greenleaf, W. J. Transposition of native chromatin for fast and sensitive epigenomic profiling of open chromatin, DNA-binding proteins and nucleosome position. *Nat. Methods* **10**, 1213–1218 (2013). 24097267 10.1038/nmeth.2688 PMC3959825

[78] Zhang, Y. et al. Model-based Analysis of ChIP-Seq (MACS). *Genome Biol.* **9**, R137 (2008). 18798982 10.1186/gb-2008-9-9-r137 PMC2592715

