# Supplementary material for: Phosphorylation of endothelial histone H3.3 serine 31 by PKN1 links flow-induced signaling to proatherogenic gene expression
Source: Nat Cardiovasc Res. 2025 Jan 8;4(2):180–96. doi: 10.1038/s44161-024-00593-y (PMC11825370; doi:10.1038/s44161-024-00593-y)

Fig. 1. f

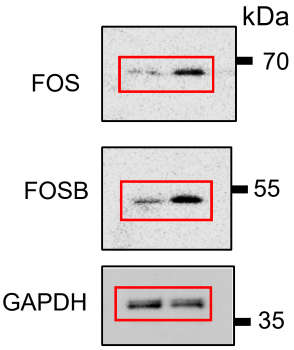

Fig. 2. b

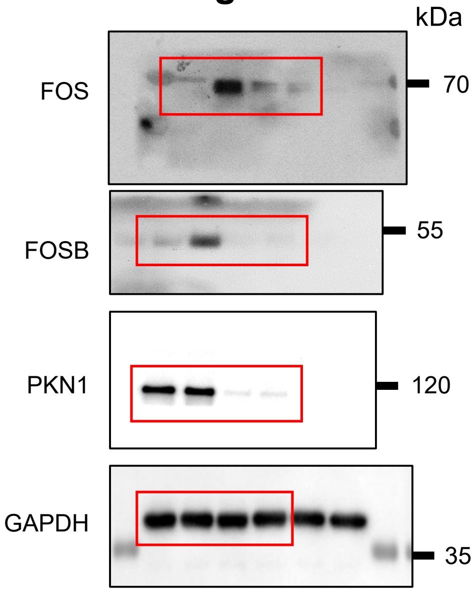

Fig. 2. d

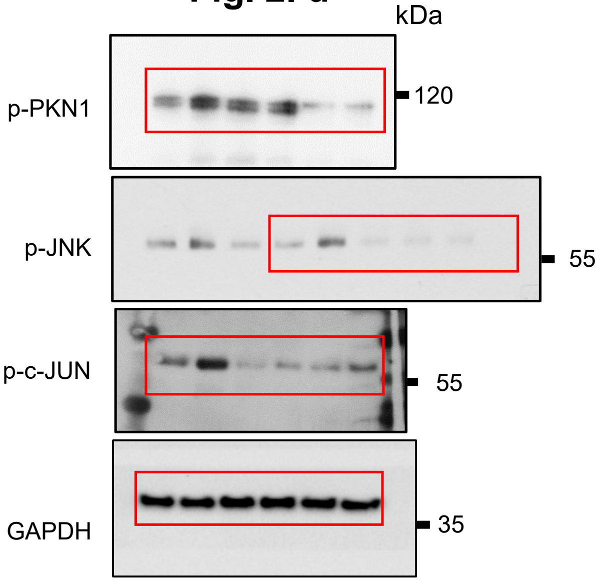

Fig. 2. h

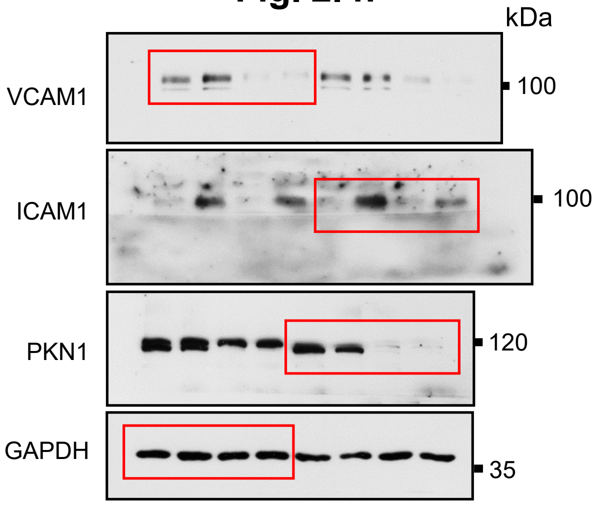

Fig. 3. a

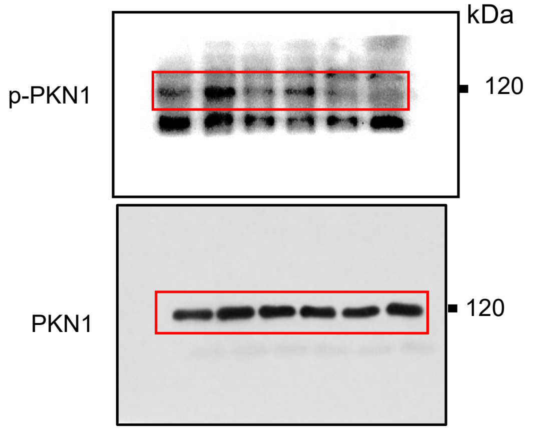

Fig. 3. b

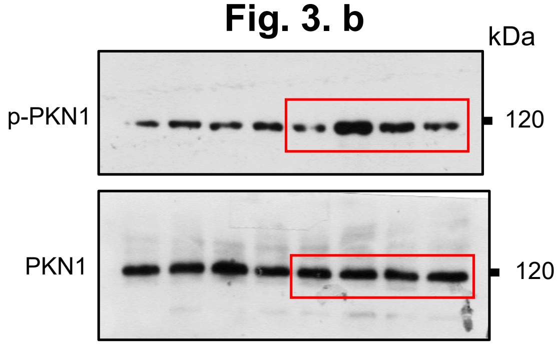

Fig. 3. d

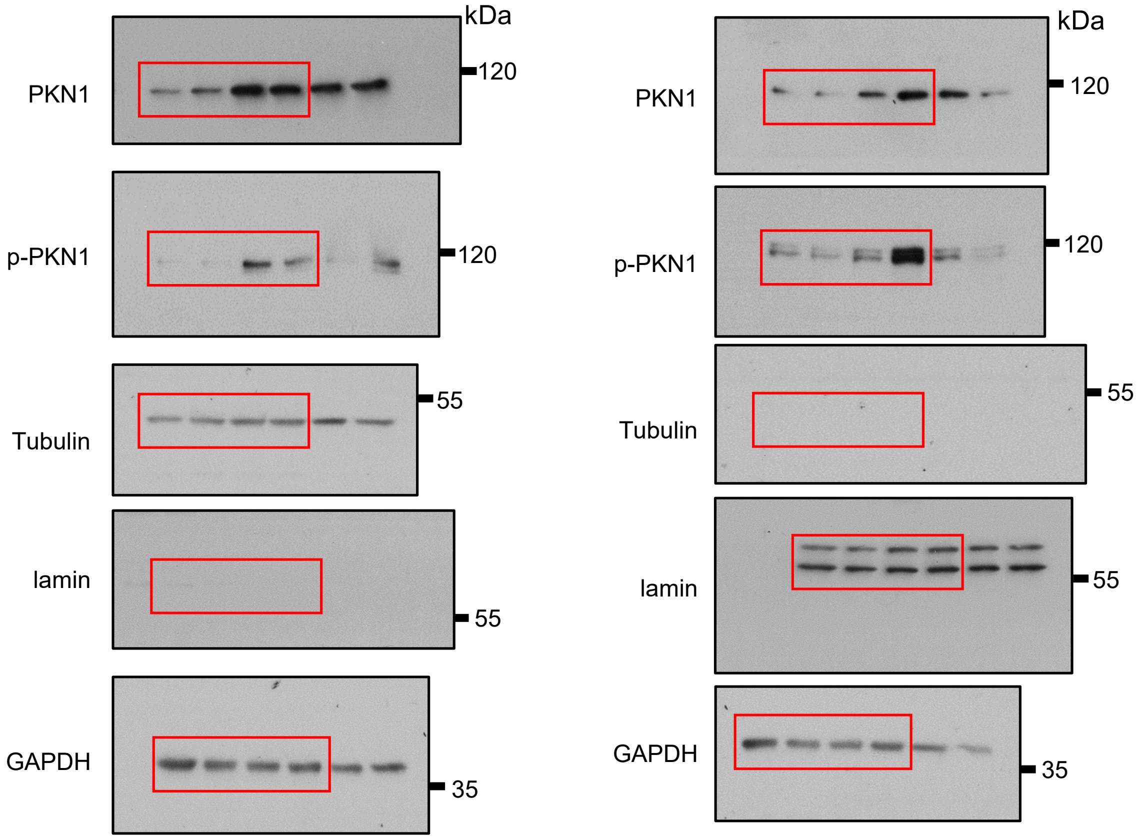

Fig. 4. a

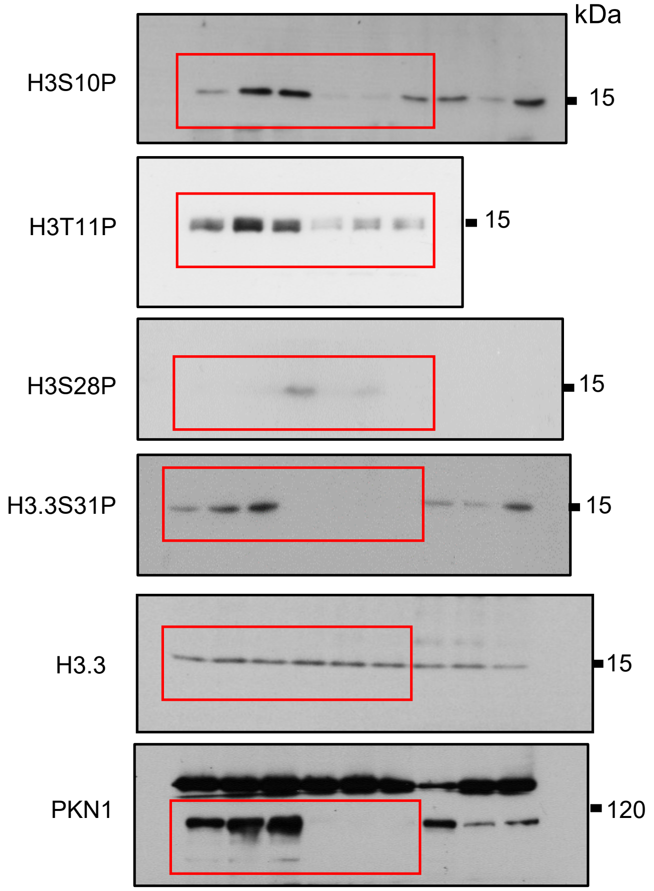

Fig. 4. f

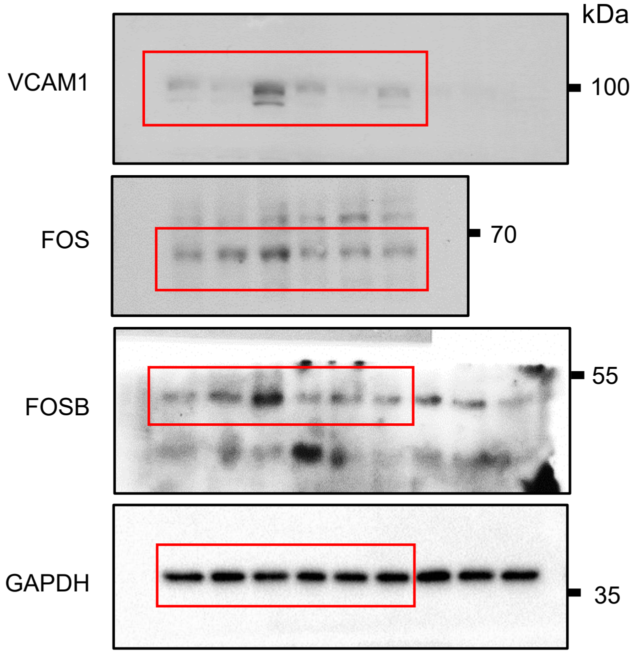

Fig. 4. g

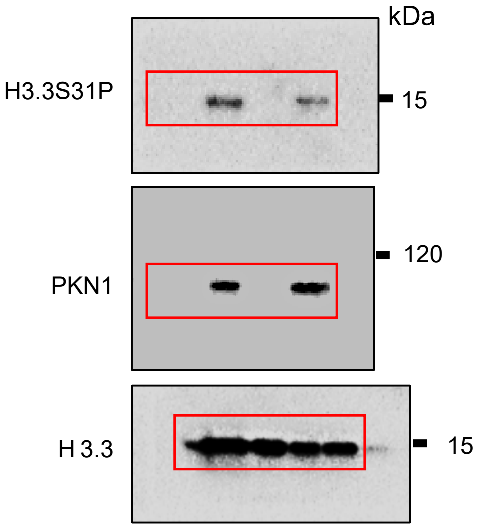

Fig. 5. a

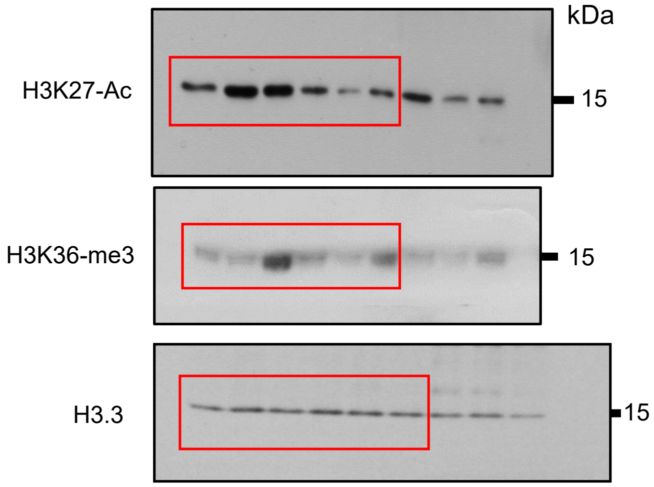
kDa
H3K27-Ac
15
H3K36-me3
15
H3.3
15

Fig. 5. b

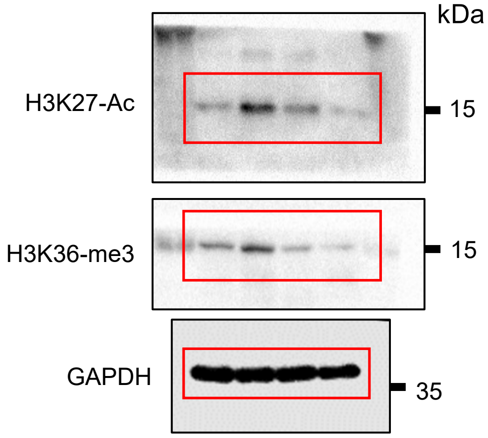
kDa
H3K27-Ac
15
H3K36-me3
15
GAPDH
35

Fig. 5. c

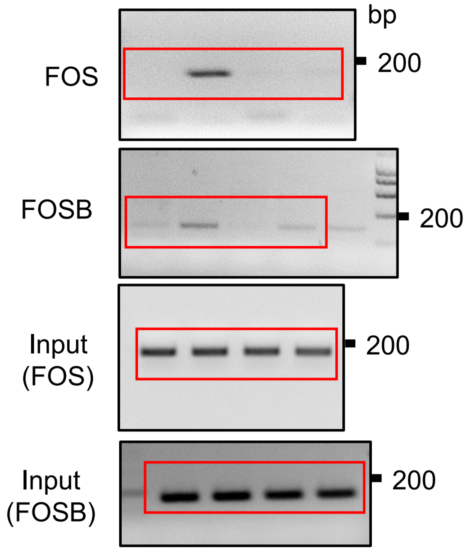

Fig. 5. d

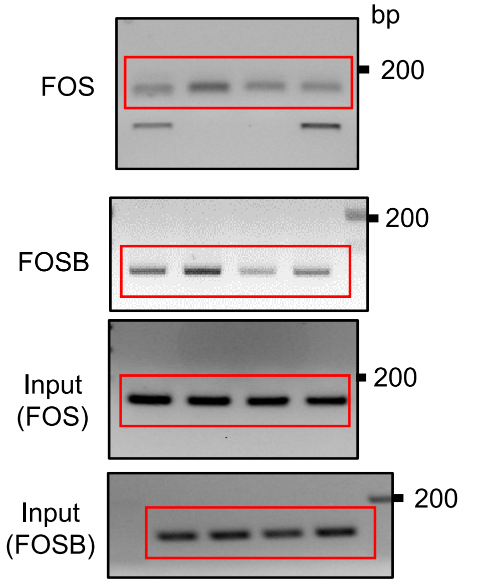

Fig. 5. e

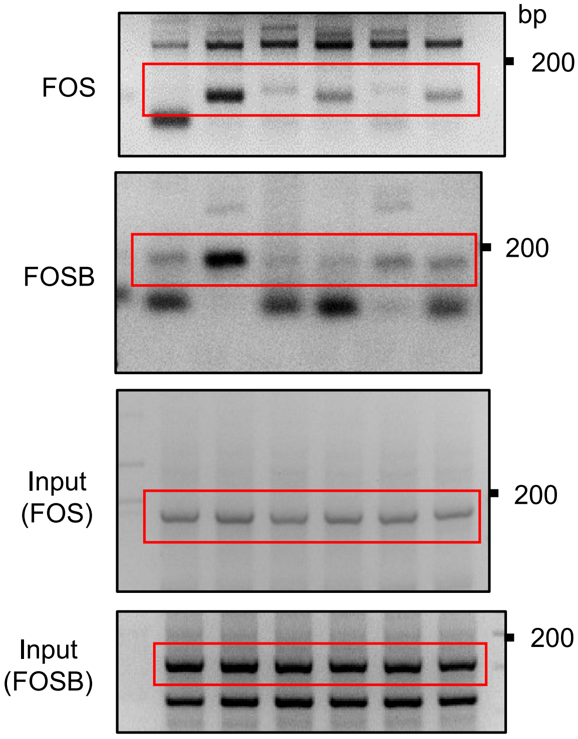

Fig. 5. f

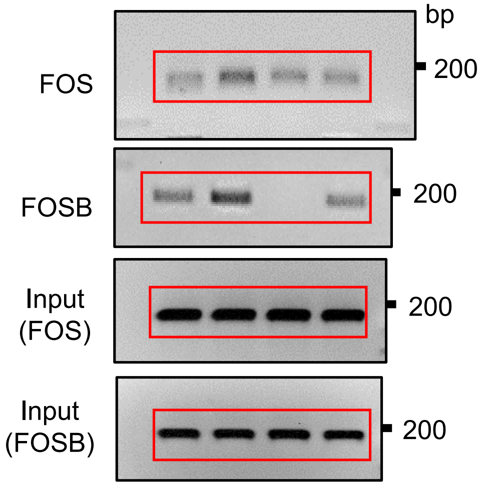

Fig. 5. g

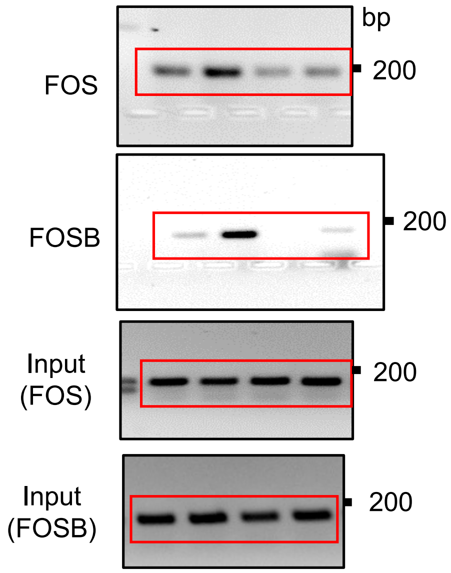

Fig. 7. a

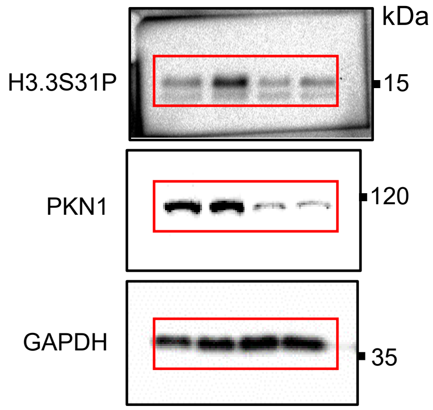
kDa
H3.3S31P
15
PKN1
120
GAPDH
35

Fig. 7. c

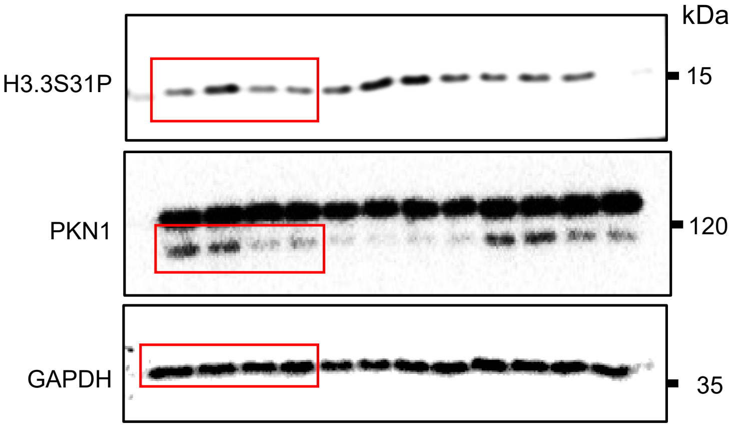
kDa
H3.3S31P
15
PKN1
120
GAPDH
35

Extended Data Fig. 1b

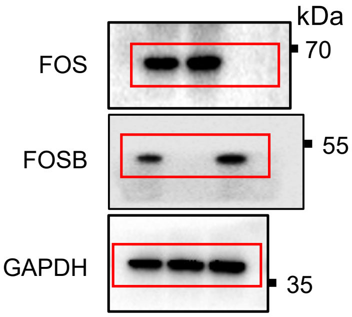

Extended Data Fig. 2e

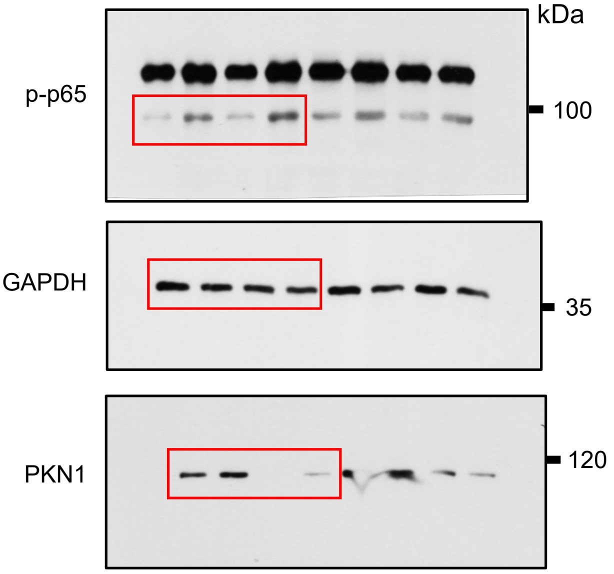

Extended Data Fig. 2g

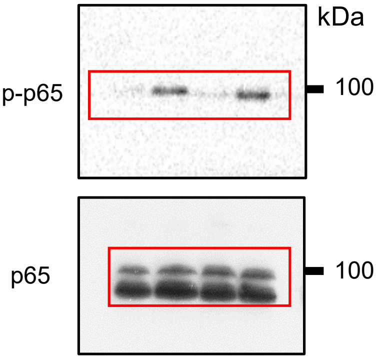

Extended Data Fig. 2h

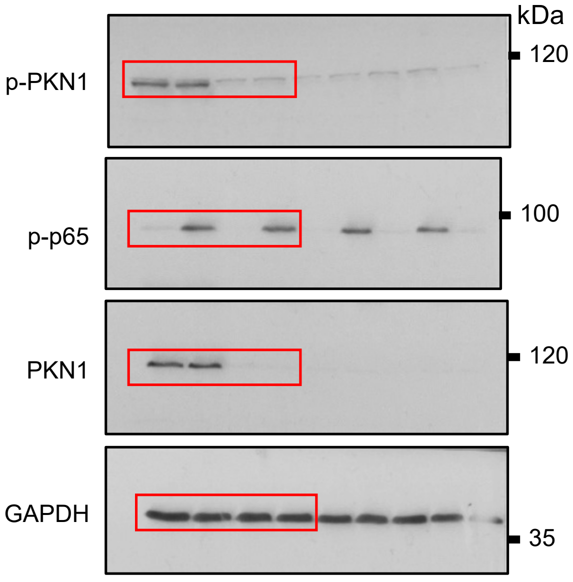

Extended Data Fig. 3a

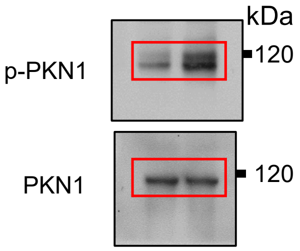

Extended Data Fig. 3b

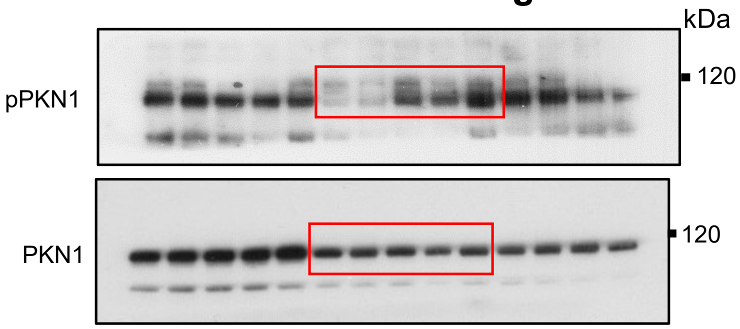

Extended Data Fig. 3c

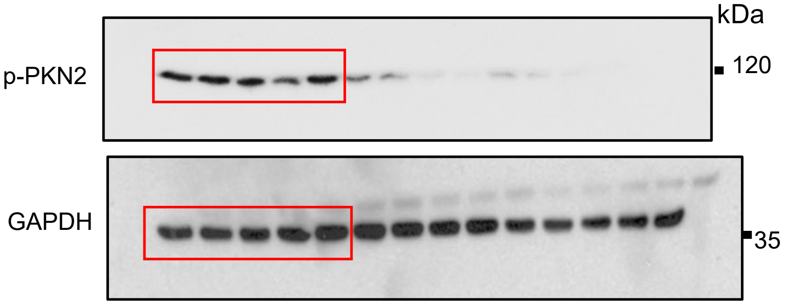

Extended Data Fig. 4c

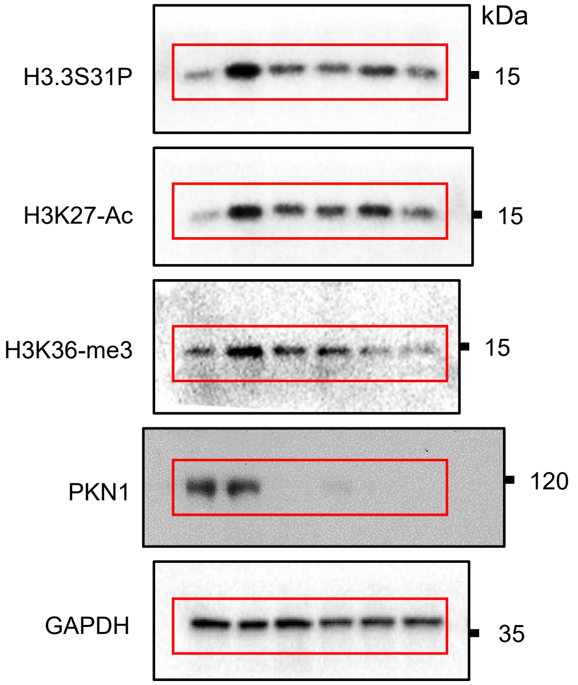

Extended Data Fig. 4e

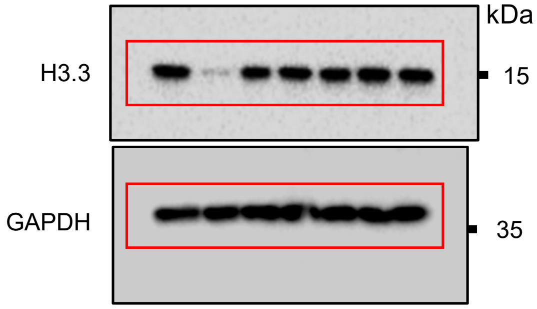

Extended Data Fig. 4d

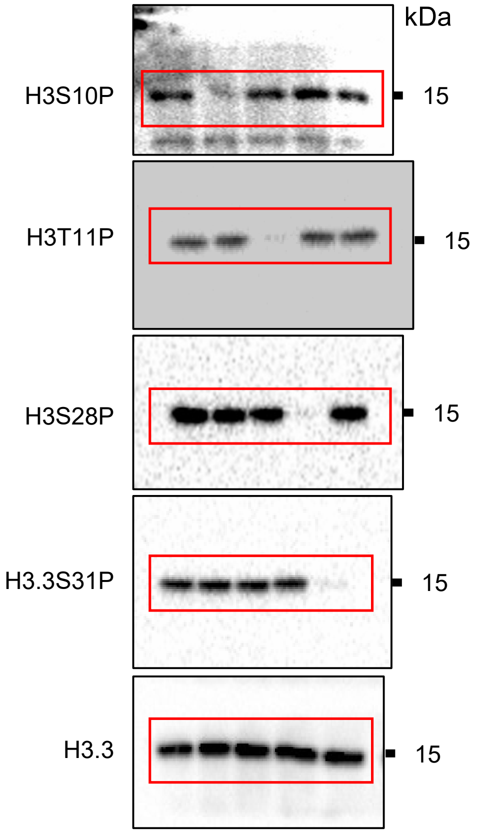

Extended Data Fig. 4f

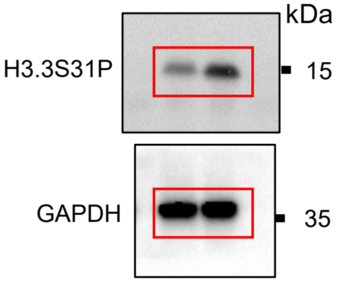

Extended Data Fig. 4g

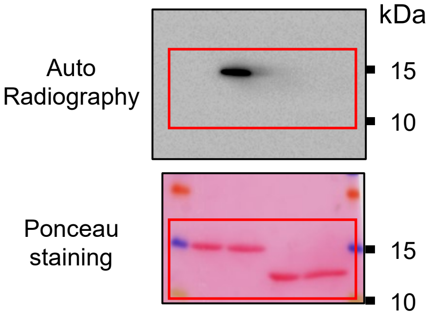

Extended Data Fig. 4h

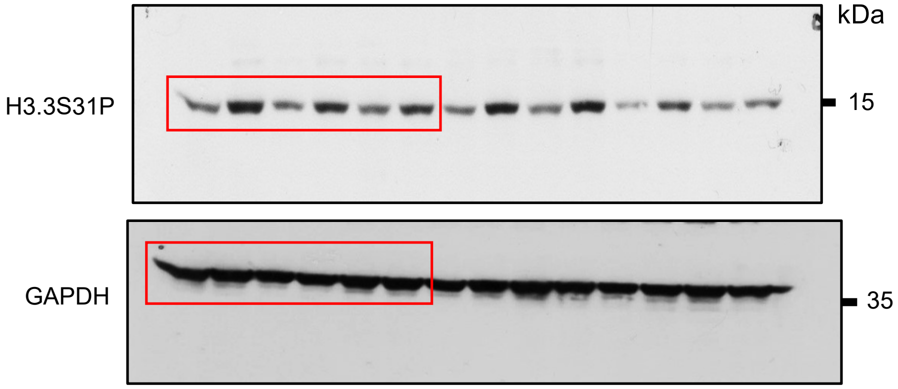

Extended Data Fig. 6a

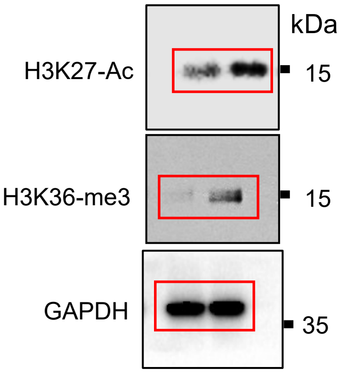

Extended Data Fig. 6b

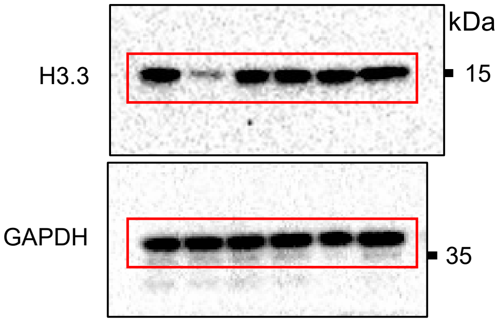

Extended Data Fig. 6d

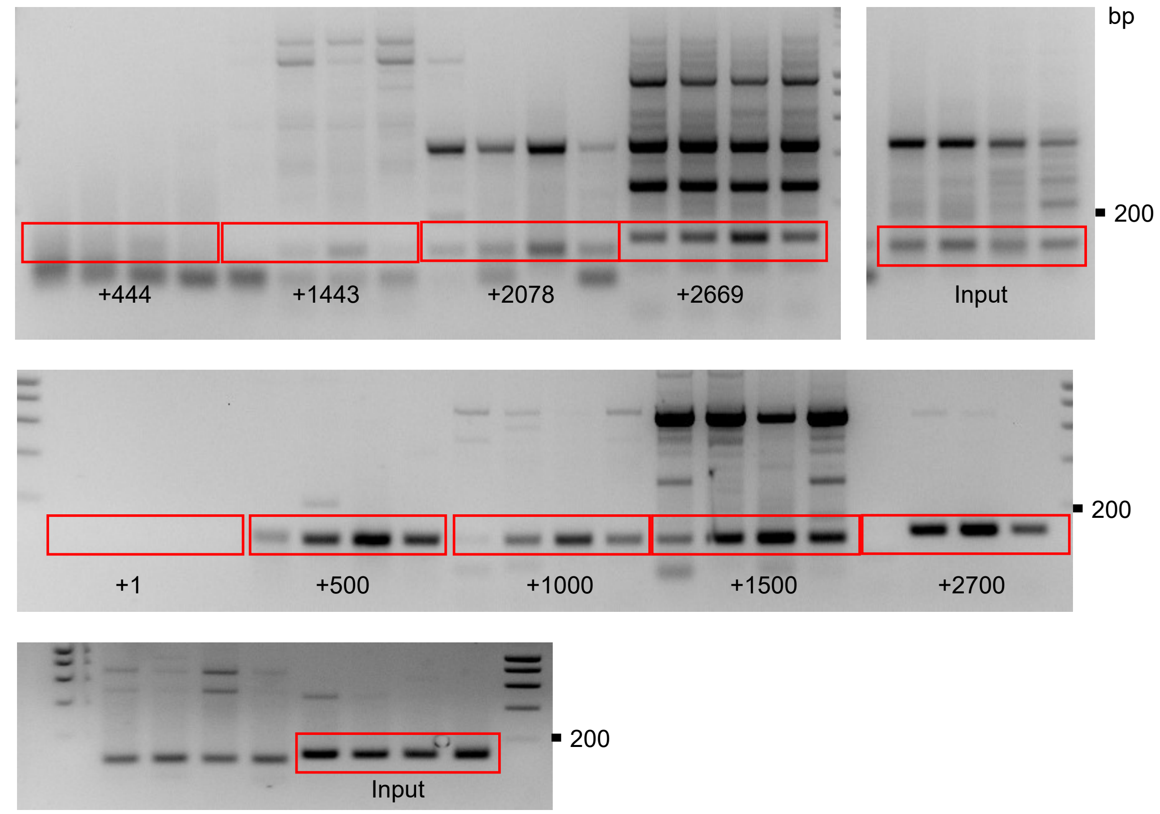

Extended Data Fig. 6e

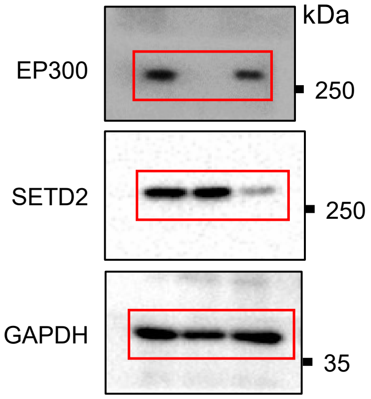

Supplement: Supplementary file 4 — Unprocessed western blots. [file 44161_2024_593_MOESM4_ESM.pdf]
